# Impact of Deltoid Computer Tomography Image Data on the Accuracy of Machine Learning Predictions of Clinical Outcomes after Anatomic and Reverse Total Shoulder Arthroplasty

**DOI:** 10.3390/jcm13051273

**Published:** 2024-02-23

**Authors:** Hamidreza Rajabzadeh-Oghaz, Vikas Kumar, David B. Berry, Anshu Singh, Bradley S. Schoch, William R. Aibinder, Bruno Gobbato, Sandrine Polakovic, Josie Elwell, Christopher P. Roche

**Affiliations:** 1 Exactech, Inc., Gainesville, FL 32653, USA; hamid.oghaz@exac.com (H.R.-O.); vikas.kumar@exac.com (V.K.); sandrine.polakovic@blue-ortho.com (S.P.); josie.elwell@exac.com (J.E.); 2 Department of Orthopedic Surgery, University of California San Diego, San Diego, CA 92093, USA; dbberry@health.ucsd.edu (D.B.B.); anshu_singh@hotmail.com (A.S.); 3 Mayo Clinic, Jacksonville, FL 32224, USA; schoch.bradley@mayo.edu; 4 Department of Orthopedic Surgery, University of Michigan, Ann Arbor, MI 48109, USA; waibinde@med.umich.edu; 5 R. José Emmendoerfer, 1449—Nova Brasília, Jaraguá do Sul 89252-278, SC, Brazil; bgobbato@gmail.com

**Keywords:** reverse total shoulder arthroplasty, anatomic total shoulder arthroplasty, machine learning, artificial intelligence, clinical decision support tools, clinical outcome predictions

## Abstract

**Background:** Despite the importance of the deltoid to shoulder biomechanics, very few studies have quantified the three-dimensional shape, size, or quality of the deltoid muscle, and no studies have correlated these measurements to clinical outcomes after anatomic (aTSA) and/or reverse (rTSA) total shoulder arthroplasty in any statistically/scientifically relevant manner. **Methods:** Preoperative computer tomography (CT) images from 1057 patients (585 female, 469 male; 799 primary rTSA and 258 primary aTSA) of a single platform shoulder arthroplasty prosthesis (Equinoxe; Exactech, Inc., Gainesville, FL) were analyzed in this study. A machine learning (ML) framework was used to segment the deltoid muscle for 1057 patients and quantify 15 different muscle characteristics, including volumetric (size, shape, etc.) and intensity-based Hounsfield (HU) measurements. These deltoid measurements were correlated to postoperative clinical outcomes and utilized as inputs to train/test ML algorithms used to predict postoperative outcomes at multiple postoperative timepoints (1 year, 2–3 years, and 3–5 years) for aTSA and rTSA. **Results:** Numerous deltoid muscle measurements were demonstrated to significantly vary with age, gender, prosthesis type, and CT image kernel; notably, normalized deltoid volume and deltoid fatty infiltration were demonstrated to be relevant to preoperative and postoperative clinical outcomes after aTSA and rTSA. Incorporating deltoid image data into the ML models improved clinical outcome prediction accuracy relative to ML algorithms without image data, particularly for the prediction of abduction and forward elevation after aTSA and rTSA. Analyzing ML feature importance facilitated rank-ordering of the deltoid image measurements relevant to aTSA and rTSA clinical outcomes. Specifically, we identified that deltoid shape flatness, normalized deltoid volume, deltoid voxel skewness, and deltoid shape sphericity were the most predictive image-based features used to predict clinical outcomes after aTSA and rTSA. Many of these deltoid measurements were found to be more predictive of aTSA and rTSA postoperative outcomes than patient demographic data, comorbidity data, and diagnosis data. **Conclusions:** While future work is required to further refine the ML models, which include additional shoulder muscles, like the rotator cuff, our results show promise that the developed ML framework can be used to evolve traditional CT-based preoperative planning software into an evidence-based ML clinical decision support tool.

## 1. Introduction

The deltoid is the largest muscle in the shoulder and the primary elevator; its size and shape power shoulder motion, particularly abduction and forward elevation. Clinical studies [1,2,3] suggest that objective measures of deltoid morphology may be prognostic of clinical performance after reverse total shoulder arthroplasty (rTSA); after all, a nonfunctioning deltoid is a contraindication for rTSA.

Despite the importance of the deltoid to shoulder biomechanics [4,5,6] and rTSA, in particular [6,7,8,9,10,11], only a few small studies have attempted to quantify deltoid muscle characteristics (e.g., size, shape, and quality) [12,13,14,15,16,17,18] and correlate [15,17,19,20] those measures to shoulder range of motion, strength, function, and patient-reported outcome measures. Several challenges have restricted previous efforts from successfully conducting muscle-to-outcomes-related research in any statistically/scientifically relevant manner. First, reliably quantifying soft tissue using various different medical imaging modalities, like magnetic resonance images (MRI) and computer tomography (CT) images, can be complex and technically challenging. Second, obtaining high-resolution imaging can be expensive, which often limits the number of patients included in these studies. Third, differences in imaging modalities and scanning protocols, including variations in image resolution and slice thickness, practically limit generalizability. Fourth, manually delineating muscle boundaries on MRI and CT images, which is necessary to obtain accurate three-dimensional (3D) muscle volumes, is tedious and time-consuming, further contributing to subjectivity in interpretations and inconsistency of measurements.

Machine learning (ML) techniques present an opportunity to objectively quantify medical images at scale. ML techniques can be used, after training, to automatically segment images to create 3D volumes of various tissues, such as bone, muscle, tendon, and fat. These segmented images can then be automatically analyzed for size, shape, and other radiomic measures [21]. Importantly, these radiomic measurements can potentially characterize tissue quality by analyzing the distribution of gray-scale voxel intensities that compose these images and 3D volumes. Hounsfield units (HUs) represent the radiation attenuation (e.g., radiodensity) of different tissues in CT images. The HU scale is defined such that the radiodensity of water has a HU value of 0, and the radiodensity of air is typically −1000 HU. Different HU ranges have been suggested for muscle and fat. Most commonly, HU intervals of −190 to −30 characterize fat, and HU intervals of −29 to 150 characterize muscle [22]. By incorporating radiomic analyses into an ML framework that includes muscle segmentation, challenges associated with traditional techniques (i.e., manual segmentation and subjective assessments) are effectively overcome. As an added advantage, because ML techniques can be efficiently scaled, larger sample sizes can be analyzed, facilitating a more granular comparative analysis with greater statistical power. 

In this study, we aim to utilize a CT-based ML framework to segment the deltoid muscle from a registry of preoperative CT images of shoulder arthroplasty patients enrolled in an IRB-approved multi-center prospective clinical outcome study. From these segmented deltoid images, we aim to quantify various muscle characteristics, including volumetric (size, shape, etc.) and intensity-based HU measurements. By analyzing these objective measures of the deltoid muscle alongside each patient’s clinical outcomes after anatomic total shoulder arthroplasty (aTSA) and rTSA, we aim to better understand the relationship of the deltoid to clinical performance. Finally, we aim to utilize these deltoid muscle image data and clinical outcomes data to create a CT-based ML model that predicts clinical outcomes after aTSA and rTSA. 

The specific goals of this study are: (1) to quantify and compare the preoperative deltoid muscle characteristics using various radiomic measurements for male and female aTSA and rTSA patients; (2) to quantify and compare the impact of varying CT image convolution kernels on these deltoid measurements; (3) to investigate if these deltoid muscle measurements correlated with preoperative pain, range of motion, and function and also investigate if these muscle measurements impact 2-year minimum postoperative clinical outcomes after aTSA and rTSA; and (4) to incorporate these deltoid muscle measurements into an ML-based predictive model, rank-order the relative importance of each image measurement to predict 2-year minimum clinical outcomes, and quantify the ability of these objective measures to improve the predictive performance of regression and classification ML models for multiple clinical outcome measures at multiple follow-up timepoints after aTSA and rTSA as compared to non-image based versions of the ML predictive models for each outcome measure. 

## 2. Materials and Methods

Preoperative CT images from 1057 patients (585 female, 469 male, and 3 unspecified; age = 70.0 ± 7.9, range: 38–92; 799 primary rTSA and 258 primary aTSA) enrolled in a multi-center, IRB-approved prospective clinical outcome study of a single platform shoulder arthroplasty prosthesis (Equinoxe; Exactech, Inc., Gainesville, FL, USA) were analyzed in this study. These patients were selected from a larger CT dataset of patients with images acquired using the ExactechGPS CT-scan acquisition protocol. This CT protocol permits slice thickness between 0.3 to 1.25 mm—with a recommended thickness of 0.625 mm, pixel resolutions between 0.3 × 0.3 mm to 1 × 1 mm, and accepts multiple different convolution kernels from different manufacturers, including but not limited to BONE (GE), B41 (Siemens), FC30 (Toshiba), and L (Philips). Each patient’s CT scan included complete acquisition of the deltoid muscle and scapular bone. 

### 2.1. Deltoid Image Analysis

**Figure 1:**
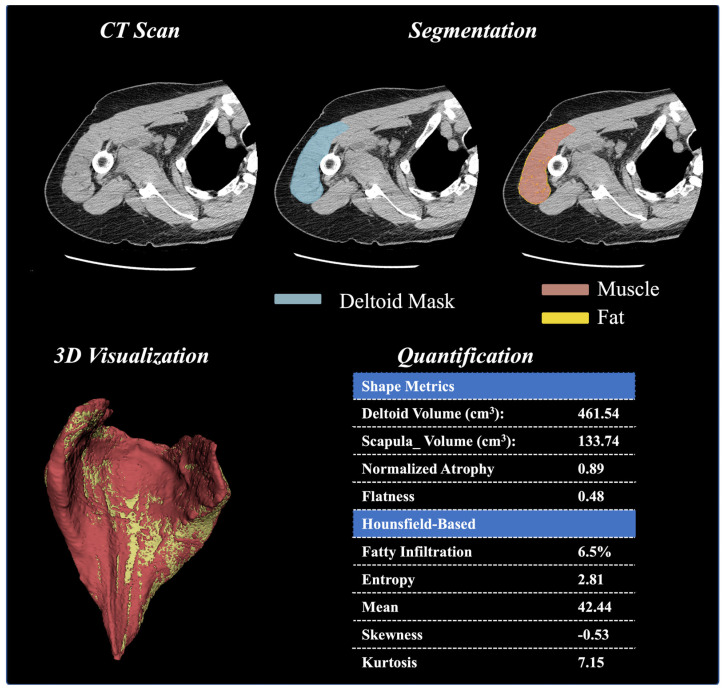
Deltoid Muscle Model Workflow, From Segmentation of CT Images to Model Creation, Volume Analysis, Hounsfield Unit Thresholding Analysis, and 3D Visualization.

An overview of the ML framework is described in Figure 1. First, a CT-based ML segmentation algorithm automatically delineates the deltoid boundaries and creates 3D deltoid masks. After deltoid segmentation, 3D models of the reconstructed deltoid were viewed by two trained evaluators to confirm that the entirety of the deltoid was present and that there were no major errors in the deltoid segmentation that could affect subsequent quantification. Cases with an estimated volumetric error >5% were excluded from further analysis.

Next, a quantification technique was utilized to extract deltoid characteristics, specifically the shape, size, and distribution of voxel intensities. The segmented masks are overlayed onto the raw images [23], and the shape and size features are calculated from the resulting mesh created by the mask and the raw image. These calculated features include deltoid volume, normalized deltoid volume (relative to the scapular bone volume), and normalized deltoid atrophy (relative to deltoids of patients having the same age and gender). Beyond these basic features, first-order radiomic shape features are derived from mesh volume and include deltoid shape flatness, sphericity, length (max 2D diameter column), and width (max 2D diameter row). In addition to these shape and size features, the first-order radiomics were calculated from the distribution of voxel intensities (i.e., distribution of HU values). These radiomic measures include skewness, entropy, uniformity, mean intensity value, root-mean-squared intensity value, 90th percentile intensity, and kurtosis. Radiomics were extracted after resampling images to an isotropic voxel of 1 mm and using a bandwidth of 25 HU. Finally, the voxels within the deltoid (segmented area) were stratified into fat and muscle based on their observed HU values (fat = −190 to −30 HU; muscle = −29 to 150 HU). Fatty infiltration was calculated as the ratio of fat (number of voxels that represent fat) to soft tissue (number of voxels that represent both muscle and fat). The definitions of each of these volumetric-based and HU intensity-based deltoid measurements are described in Table 1. 

The ML CT image segmentation algorithm used in this study to automatically segment the deltoid has been previously validated [24]. This deltoid segmentation algorithm was fine-tuned from a pre-trained model using SwinUNETR. The SwinUNETR model is a U-Net architecture that uses Swin transformers instead of convolutional neural networks (CNNs) to extract features. Swin transformers are a type of attention-based neural network architecture that effectively capture long-range dependencies in data, making them well-suited for tasks such as medical image segmentation [25]. The model was fine-tuned using CT scans from 78 patients, from which medical students, under the supervision of an orthopedic surgeon and experienced radiologist, manually delineated the deltoid boundaries on each CT image to generate labeled masks. After training using these masks, the model was deployed and tested on CT images from an additional 20 patients and was demonstrated to produce deltoid masks with a high dice coefficient of 0.93 ± 0.03 [24].

### 2.2. Predict+ Background

Predict+ (Exactech, Inc., Gainesville, FL, USA) is an ML-based clinical decision support tool (CDST) that preoperatively predicts personalized aTSA and rTSA outcomes from a “minimal feature set” of preoperative inputs [26,27]. Specifically, ML-based regression predictions for 7 outcome measures (VAS pain, global shoulder function, shoulder arthroplasty smart (SAS) score [28], active abduction, active forward elevation, active external rotation, and internal rotation (IR) score [29]) are made at 6 postoperative timepoints (3–6 months, 6–9 months, 1 year, 2–3 years, 3–5 years, and 5+ years). With additional preoperative inputs, the ASES and Constant score can also be predicted at the same timepoints. Classification predictions are provided 2–3 years after surgery to describe the likelihood that a patient will achieve improvement that exceeds the minimal clinically important difference (MCID) [28,30,31] and substantial clinical benefit (SCB) [28,31,32] patient-satisfaction thresholds associated with each outcome measure. The internal validations describing the accuracy of the Predict+ ML algorithms are published [26,31,33,34,35,36,37], and these algorithms have also been externally validated [27] for up to 2 years. Furthermore, recent research has demonstrated that Predict+ provides fair [36] and unbiased predictions for aTSA and rTSA patients of different ethnicities, sexes, and ages. Additionally, the use of Predict+ has been demonstrated to improve surgeon confidence [38] when deciding between aTSA and rTSA. 

Currently, the Predict+ “minimal feature set” [26] of preoperative input requirements does not include any direct CT image data or any data related to the quality of a patient’s soft tissue or bone. Due to the widespread clinical use of CT-based preoperative planning software tools for both aTSA and rTSA, ample CT data are readily available in existing clinical workflows to enhance CDST predictions and further support clinical decision-making by including each patient’s CT image information related to their bones and soft tissue. However, it is currently unknown which features derived from CT image data are useful to improve the accuracy of the ML-based CDST outcome predictions. 

### 2.3. Analysis of Deltoid Image Measurements and Clinical Outcomes

To better understand the relationship between CT-based deltoid image measurements and clinical outcomes after aTSA and rTSA, preoperative and postoperative clinical data from 1057 primary shoulder arthroplasty patients were analyzed with each patient’s preoperative CT-based deltoid image data. Preoperative outcome measures and 2-year minimum clinical outcomes were compared with numerous different deltoid image measurements for various patient cohorts and were statistically analyzed using a two-tailed unpaired students *t*-test, with a *p*-value < 0.05 defining significance. Additionally, a Pearson correlation analysis was performed to measure the strength and direction of linear associations between the deltoid image measurements and preoperative clinical measures and between deltoid image measurements and 2-year minimum clinical outcomes.

### 2.4. Machine Learning Prediction

XGBoost is a supervised, ensemble ML technique of multiple-regression trees that are built by iteratively partitioning the training dataset into multiple small batches using a method called boosting [39,40]. XGBoost was used to construct algorithms that predict 1-year (9–18 months), 2–3-year (18–36 months), and 3–5-year (36–60 months) aTSA and rTSA outcomes for each aforementioned clinical outcome measure both before and after addition of the CT image deltoid data. Specifically, two ML models were constructed: (1) non-image-based model (which utilizes the minimal feature set of inputs + implant data and bone measurements readily available in CT planning software, e.g., ExactechGPS Equinoxe Planning App v2) and (2) image-based model (which utilizes all of the inputs form the first model + the CT image based deltoid measurements). 

The clinical data from 258 primary aTSA patients (average follow-up = 27.1 months) and 799 primary rTSA patients (average follow-up = 26.1 months) was used to build and test predictive models at each postoperative timepoint: 1 year (aTSA = 152 and rTSA = 402 visits), 2–3 years (aTSA = 178 and rTSA = 533 visits), and 3–5 years (aTSA = 78 and rTSA = 201 visits). Similar to the methodology of the Predict+ internal validations [26,31,33,34,35,36,37], the dataset was randomly split into 70%:30% mutually exclusive datasets to build and test the predictive models using each of the image-based and non-image-based input datasets for each clinical outcome metric at the 1-year, 2–3-year, and 3–5-year timepoints. The process is repeated using k-fold cross-validations (*n* = 5) to reduce any overfitting in the model.

The predictive performance of each regression model prediction was quantified by the Mean Absolute Error (MAE), which describes the mean absolute difference between the actual and predicted values of each clinical outcome measure at each of the 1-year, 2–3-year, and 3–5-year timepoints. The predictive performance of each 2–3-year MCID [28,30,31] and SCB [28,31,32] classification model describing if a patient will achieve clinical improvement that exceeds the MCID and SCB improvement thresholds was quantified using the classification metrics of accuracy (which quantifies the ability of a model to correctly predict a class), precision or positive predictive value (which quantifies the ability of a model to not identify a negative as positive), recall or sensitivity (which quantifies the ability of a model to identify a positive as a positive), and the area under the receiver operating curve (AUROC). 

The relative importance of each preoperative input data to predict each 2–3-year clinical outcome measure was quantified by the F-score and the Reciprocal Fusion Rank score. The F-score is determined by the XGBoost ML technique and quantifies the predictive value of an individual feature to the overall algorithm by the frequency that each feature is used as a candidate for the split by the decision tree algorithm [40]. The Reciprocal Fusion Rank score [41] combines the F-score value with the prevalence and uniqueness of that feature within the dataset, deprioritizing features with non-unique and sparse inputs [40,41]. Combined, these feature importance data are useful for interpretability [42,43] to better understand the internal logic of the ML model and review the basis of the predictions, which can be particularly important when evaluating radiomic features as some of these measurements are non-intuitive. 

Finally, to investigate the feasibility of constructing an ML clinical outcome model that does not require manual input of preoperative range of motion data or patient subjective pain and function survey responses (to improve ML CDST usability and minimize responder fatigue), we constructed a new ML model to predict clinical outcomes after aTSA and rTSA at 1 year, 2–3 years, and 3–5 years by substituting the deltoid image data for the aforementioned surgeon-measured objective range of motion data and patient subjective survey data. We then quantified the predictive accuracy of this new image-based ML model (which effectively simulates inputs that can be automatically obtained from CT-based preoperative planning software) and compared this predictive accuracy to the non-image-based ML models for each outcome measure at each postoperative timepoint. 

## 3. Results

The results of this study are presented in three sections. First, we present the deltoid shape, size, and radiomic data for the 1057 shoulder arthroplasty patients and provide a statistical analysis describing the relationship of these muscle measurements to patient demographics and clinical outcomes after aTSA and rTSA. Second, we present the impact of deltoid fatty infiltration and the impact of convolution kernel on clinical outcomes after aTSA and rTSA. Third, we present the predictive accuracy associated with the Predict+ ML models utilizing the deltoid image data and report on the feature importance rankings of each deltoid measurement to predict clinical outcomes after aTSA and rTSA.

### 3.1. Deltoid Shape, Size, and Radiomics

The ML framework successfully segmented and extracted radiomic data, including volumetric and HU intensity-based measurements from the CT images of 1057 patients without any manual correction. Substantial variability in CT-based deltoid measurements was observed between patient cohorts when stratified by gender, prosthesis type (Table 2), and by convolution kernel (Table 3). As described in Table 2, significant differences by gender and prosthesis type were observed for four of the six mean volumetric deltoid measurements (deltoid shape flatness, normalized deltoid volume, max column, and max row). Additionally, significant differences by gender and prosthesis type were observed for five of the eight mean HU intensity deltoid measurements (deltoid fat %, skewness, mean voxel, root mean square voxel, and 90th percentile voxel). No significant differences in the mean radiomic measurements for normalized deltoid atrophy, entropy, uniformity, or kurtosis were observed between male and female patients for aTSA or rTSA. However, significant differences were observed in mean deltoid measurements when stratified by convolution kernel for every measurement except deltoid shape flatness and kurtosis. 

The mean deltoid volume of the 1057 patients in our study was 342.2 ± 113.0 cm^3^. Mean deltoid volume was observed to decline with patient age by approximately 6% per decade for male patients and 8% per decade for female patients. As described in Table 4, these trends were observed for the direct deltoid volume measurement and the deltoid volume measurements when normalized by scapular bone volume and patient height. Normalized deltoid volume (by scapular bone volume) demonstrated greater relevance to preoperative clinical measures (Table 5) than 2-year minimum postoperative clinical outcome measures (Table 6) after aTSA and rTSA. Prior to surgery, larger normalized deltoid volume was associated with a significantly higher SAS score, significantly more abduction, forward elevation, and internal rotation for male patients and significantly more abduction for female patients. At a minimum of 2 years after aTSA, no differences were observed in any clinical outcome measure between patients with a normalized deltoid volume <4 and patients with a normalized deltoid volume >4. However, some 2-year minimum postoperative differences were observed with rTSA. Male rTSA patients with larger normalized deltoid volumes had significantly higher VAS pain, whereas female rTSA patients with larger normalized deltoid volumes had significantly more abduction but significantly less global shoulder function and significantly lower ASES and SAS scores. 

### 3.2. Fatty Infiltration and the Impact of Convolution Kernel

**Figure 2:**
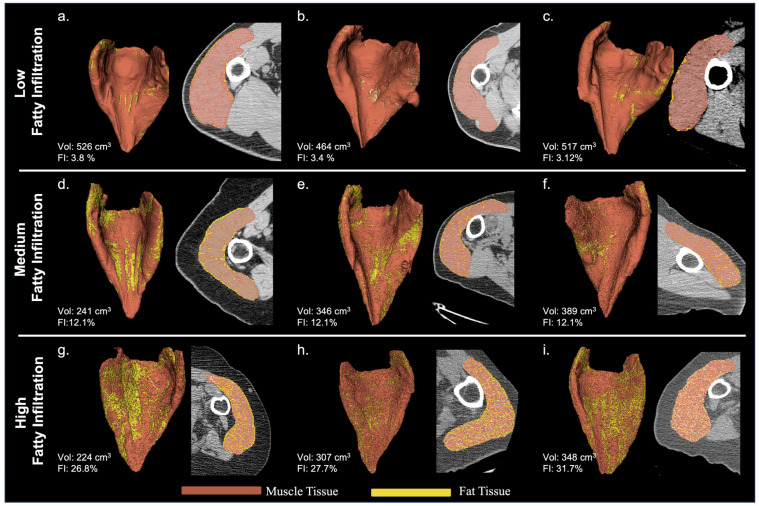
Representative Deltoid Muscle with Low ((**a**–**c**): **top row**), Medium ((**d**–**f**): **middle row**), and High ((**g**–**i**): **bottom row**) Levels of Fatty Infiltration for 3D Volumes Reconstructed from BONE Kernel CT Images.

**Figure 3:**
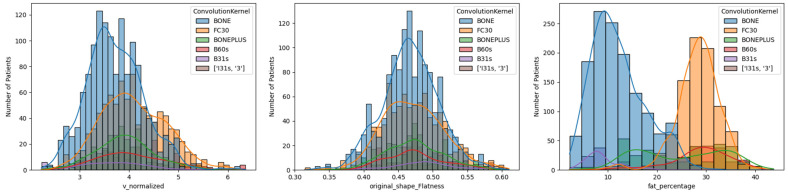
Histogram Comparison of Normalized Deltoid Volume (**left**), Deltoid Flatness (**middle**), and Deltoid Muscle Fatty Infiltration Percentages (**right**) for Different CT Convolution Kernels. Note the difference in distribution for the Hounsfield thresholding-based measurements of Deltoid fat percentage between BONE and FC30, which are the two most common convolution kernels for CT images in this study.

A wide range of deltoid fatty infiltration was observed across patient cohorts. As illustrated in Figure 2, patients with lower deltoid fat percentages tended to have fat along the periphery of the muscle, whereas patients with higher fat percentages tended to have fat distributed throughout the muscle. Considering all convolution kernels, the mean deltoid fat percentage was 20.0 ± 9.9%, though deltoid fat percentage varied on average from 18 to 20% for male patients and from 19 to 23% for female patients across the age cohorts (Table 4). Importantly, the mean deltoid fat percentage was significantly different between male and female patients for both aTSA and rTSA (Table 3) and the mean deltoid fat measurements varied significantly by convolution kernel (Table 3 and Figure 3), particularly between the BONE kernel and the FC30 kernel (Figure 3). 

Interestingly, the relationship between deltoid fatty infiltration and patient demographics and comorbidities varied by convolution kernel. As described in Table 7, for BONE kernel CT images, male patients with greater deltoid fatty infiltration had significantly higher BMI and more comorbidities; in particular, a significantly greater percentage of fatty infiltration patients had diabetes. Similarly, for BONE kernel CT images, female patients with greater deltoid fatty infiltration had significantly higher weight, higher BMI, a lower occurrence of CTA, and more comorbidities; in particular, a significantly greater percentage of fatty infiltration patients had inflammatory arthritis, hypertension, and diabetes. In contrast, for FC30 kernel CT images, male patients with greater deltoid fatty infiltration had a significantly lower weight but had significantly more comorbidities. Similarly, for FC30 kernel CT images, female patients with greater deltoid fatty infiltration had significantly more comorbidities. 

Deltoid fatty infiltration was demonstrated to have some impact on both preoperative clinical measures (Table 8) and 2-year minimum postoperative clinical outcome measures (Table 9) after aTSA and rTSA. However, this impact varied by gender and by convolution kernel, particularly preoperatively. Prior to surgery, male patients with greater deltoid fatty infiltration had significantly less deltoid volume (for both the BONE and FC30 kernel cohorts), and specifically for FC30 kernel CT images, male patients with high fatty infiltration also had significantly less forward elevation and less strength (as measured by the max weight held in hand measurement from the Constant score). Prior to surgery, female patients with greater fatty infiltration, specifically for BONE kernel CT images, had significantly larger deltoid volumes, more abduction, less internal rotation, more external rotation, more strength, and a higher global shoulder function score; in contrast, for FC30 kernel CT images, females with greater fatty infiltration had a significantly lower SAS score. At a minimum of 2 years after aTSA, patients with greater fatty infiltration had significantly less strength and a significantly lower Constant score (for both the BONE and FC30 kernel cohorts). Specifically for BONE kernel CT images, aTSA patients with greater fatty infiltration also had significantly less internal rotation. Specifically, for FC30 kernel CT images, aTSA patients with greater fatty infiltration also had significantly less abduction. At a minimum of 2 years after rTSA, patients with greater fatty infiltration had significantly less strength and significantly lower Constant and ASES scores (for both the BONE and FC30 kernel cohorts). Specifically, for BONE kernel CT images, rTSA patients with greater fatty infiltration had significantly less forward elevation and internal rotation and a significantly lower SAS score. 

### 3.3. Deltoid Features in Predict+

Both image-based (using deltoid characteristics) and non-image-based XGBoost ML regression models resulted in accurate clinical outcome predictions after aTSA and rTSA (Table 10). The addition of deltoid image data to the 1-year, 2–3-year, and 3–5-year XGBoost ML regression models resulted in modest improvements in predictive accuracy for most outcome measures relative to the ML regression models without image data (Table 10). The largest improvements in predictive accuracy were observed for the active abduction and forward elevation ML regression models. Specifically, for active abduction, the deltoid image-based ML models were associated with lower MAE for both aTSA and rTSA at each timepoint, with the most substantial improvement being a 16.1% reduction in MAE for aTSA at 3–5 years as compared to the ML models without image data. Similarly, for forward elevation, the deltoid image-based ML models were associated with lower MAE for both aTSA and rTSA at each timepoint, with the most substantial improvement being a 10.8% reduction in MAE for aTSA at 2–3 years as compared to the ML models without image data. Some marginal differences in predictive accuracy were also observed in the other outcome measure ML models.

The MCID (Table 11) and SCB (Table 12) classification predictions associated with each of the image- and non-image-based clinical outcome ML models at 2–3 years follow-up are presented in Table 11 and Table 12, respectively. Both image-based and non-image-based XGBoost ML classification models resulted in accurate MCID and SCB predictions after aTSA and rTSA, with very little improvement observed by the addition of the deltoid image data. For aTSA patients, the deltoid image-based predictive models achieved 82–93% accuracy in MCID with an AUROC between 0.69–0.80 and 77–91% accuracy in SCB with an AUROC between 0.70–0.91. For rTSA patients, the deltoid image-based predictive models achieved 77–94% accuracy in MCID with an AUROC between 0.69–0.85 and 74–91% accuracy in SCB with an AUROC between 0.72–0.86.

**Figure 4:**
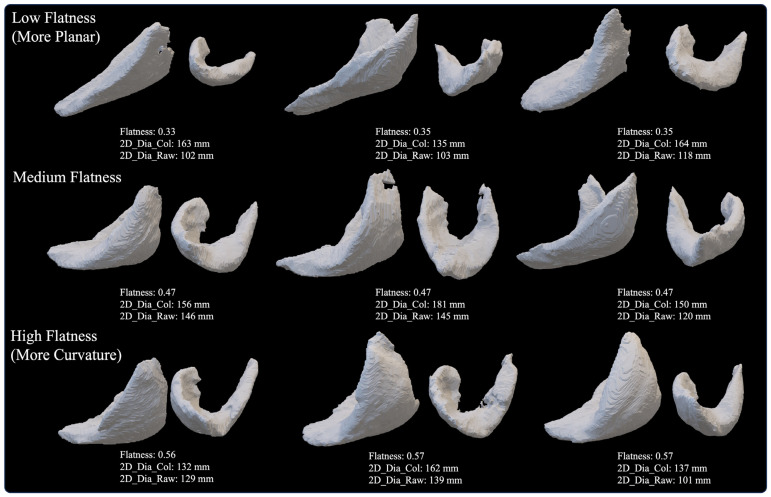
Representative Deltoid Muscle with Low (**top row**, more planar), Medium (**middle row**), and High (**bottom row**, more curvature) Values of Deltoid Shape Flatness for 3D Volumes Reconstructed from BONE Kernel CT Images.

The F-scores and Reciprocal Fusion Rank scores describing the relative feature importance ranking of each CT-based deltoid image parameter to predict 2–3-year clinical outcomes after aTSA and rTSA are presented in Table 13. A review of these F-scores and Reciprocal Fusion Rank score rankings demonstrates that eight out of fourteen deltoid image measurements analyzed in this study were of high predictive value to each 2–3-year clinical outcome model. Deltoid shape flatness was the consensus most predictive deltoid image measurement, being the most utilized feature to predict abduction, external rotation, IR score, ASES, and SAS scores. Deltoid shape flatness was also the second most utilized feature to predict the Constant and VAS pain scores. For reference, deltoids associated with low, medium, and high flatness are depicted in Figure 4. Generally, deltoids with low flatness values were more planar, which may suggest smaller anterior and posterior deltoids (and potentially smaller moment arms), while deltoids with higher flatness values tend to be more curved and/or spherically shaped, with potentially larger anterior and posterior deltoids (and potentially larger moment arms). Normalized deltoid volume was the second most utilized deltoid image measurement and was found to be the most utilized feature to predict the Constant and global shoulder function scores. Other important deltoid image measurements in order of feature importance are deltoid voxel skewness, deltoid shape sphericity, normalized deltoid atrophy, deltoid fat percentage, deltoid voxel entropy, and deltoid voxel uniformity. For additional context as to which preoperative data are used to predict 2–3-year outcomes with each ML model, the features with the top 10 F-scores for each outcome prediction are presented in Table 14. As described in Table 14, preoperative abduction and native glenoid retroversion were generally the most predictive features used to predict 2–3-year outcomes after aTSA and rTSA. 

Interestingly, despite the high predictive value for each aforementioned feature to predict 2–3-year clinical outcomes, Pearson correlation analysis (Table 15) demonstrated a low linear correlation between these deltoid measurements and each preoperative and 2-year minimum clinical outcome measure for both aTSA and rTSA. Additionally, above- or below-average deltoid shape flatness, being the overall most predictive feature across all ML models, was observed to only modestly discriminate between patient cohorts for mean preoperative and postoperative outcome measures (Table 16 and Table 17). Prior to surgery, male patients with deltoid flatness >0.47 had significantly more internal rotation than male patients with deltoid flatness <0.47, whereas female patients with deltoid flatness >0.47 had significantly more internal rotation, significantly less pain, and significantly higher ASES and SAS scores as compared to female patients with deltoid flatness <0.47 (Table 16). At a minimum of 2 years after aTSA, no differences were observed in any clinical outcome measure between patients with deltoid flatness >0.47 and patients with deltoid flatness <0.47 (Table 17). However, some 2-year minimum postoperative differences were observed with rTSA. Male rTSA patients with deltoid flatness >0.47 had significantly less strength and significantly lower Constant scores as compared to male patients with deltoid flatness <0.47, whereas female rTSA patients with deltoid flatness >0.47 had significantly less strength as compared to female patients with deltoid flatness <0.47. 

Table 18 presents the MAE associated with the alternative deltoid image ML model (which excluded manual inputs of preoperative range of motion and patient subjective pain/function survey questions) compared to the MAE for the non-image-based ML model to predict 1-year, 2–3-year, and 3–5-year clinical outcomes after aTSA and rTSA. As presented in Table 18, similar MAEs were observed between each ML model for each timepoint. Despite not including any preoperative motion measurements for abduction, forward elevation, external rotation, or internal rotation as ML model inputs and not including preoperative values for VAS pain, global shoulder function, ASES, Constant, or SAS scores as ML model inputs, including the preoperative questions that are used to score each of these measures, only a few MAEs exceeded 10% difference between the two clinical outcome prediction models across each timepoint for both aTSA and rTSA. Though generally, smaller MAE differences between models were observed for rTSA as compared to aTSA. 

## 4. Discussion

The results of this 1057 patient study demonstrate that our CT-based ML framework can automatically segment the deltoid from preoperative CT scans utilized for preoperative planning software and then automatically quantify numerous volumetric-based and HU intensity-based deltoid measures from those segmented images. An analysis of these radiomic features demonstrated that several deltoid muscle measurements vary with age, gender, prosthesis type, and CT image kernel; additionally, many of these deltoid measurements (like normalized deltoid volume and deltoid fatty infiltration) were demonstrated to be relevant to preoperative and postoperative clinical outcomes after aTSA and rTSA. Additionally, we constructed ML models using preoperative CT-based deltoid image data and demonstrated that the addition of these image data improves ML model performance, with the largest improvements in accuracy observed for the prediction of abduction and forward elevation after aTSA and rTSA. Finally, we rank-ordered the input features driving those ML models and identified the specific deltoid measurements, as well as the top 10 preoperative features that were most predictive of postoperative clinical outcomes after aTSA and rTSA. In particular, we identified that deltoid shape flatness, normalized deltoid volume, deltoid voxel skewness, and deltoid shape sphericity were the consensus most predictive image-based features used to predict clinical outcomes after aTSA and rTSA. Many of these deltoid measurements were found to be more predictive of aTSA and rTSA postoperative outcomes than patient demographic data, comorbidity data, and diagnosis data. 

This study is the largest of its kind to analyze deltoid muscle morphology and correlate those objective image-based measures of deltoid size/shape and muscle quality to clinical outcomes after aTSA and rTSA. This research is also significant because it illustrates how the application of an ML-based framework can evolve traditional CT-based preoperative planning software into an evidence-based ML-CDST. Over the past decade, CT-based preoperative planning software has become widely adopted for worldwide use with shoulder arthroplasty. This software helps surgeons better appreciate a patient’s bony morphology and/or deformity of the scapula and/or humerus, facilitating personalized implant type/size selection and identification of the precise implant position that best fits a patient’s bone while avoiding impingement. However, the ideal placement of any implant for any bony deformity is currently unknown, and despite 10+ years of clinical use of preoperative planning software with shoulder arthroplasty, no consensus guidelines exist for how to utilize these tools to optimally position implants for various bony deformities [44,45]. Effectively, the current state only facilitates surgeons to shape match a virtual implant model to a bone model however they think best. Furthermore, no preoperative planning software currently provides visualization and/or analysis of a patient’s muscles; therefore, the use of these tools and any heuristic derived from their use has been exclusively based only on bone visualization. Our study, using the deltoid as an example, demonstrates that an ML framework can, at scale, automatically segment and analyze muscles from preoperative CT images and then input that objective muscle data into an ML-based model that more accurately predicts postoperative clinical outcomes after aTSA and rTSA. Deployment of this ML framework within CT-based preoperative planning software can facilitate a more quantitative assessment of the shoulder that can be helpful to better characterize a patient’s diagnosis/pathology on a continuum, as opposed to just a subjective classification. We demonstrated that these deltoid measurements are correlated to and highly predictive of clinical outcomes after aTSA and rTSA, and because these measurements are some of the most commonly used and important features driving each ML clinical outcome model, we demonstrated that these automated CT image-based measurements can be used as a substitute for the “minimal feature set” of manual inputs (i.e., surgeon-measured preoperative range of motion data and patient subjective survey questions related to pain and function) without sacrificing ML predictive accuracy. As such, this framework, when integrated into the CT planning software, effectively creates an automated personalized ML-CDST, requiring little-to-no manual input. 

The strength of our study is the large scale of our CT analysis, which quantified numerous radiomic measurements of deltoids from 1057 patients. Nearly all the clinical literature related to the analysis of shoulder muscles has limited generalizability and limited statistical power due to small sample sizes (n~100 or less). Additionally, due to the complexity and technical challenge of quantifying deltoid muscle characteristics from medical imaging, the results of many of these studies are further limited due to the simplified 2D methodologies that they deployed to characterize the 3D deltoid morphology and/or methodologies, which only analyze a small portion of the muscle in 3D [14,15,16,17,18]. Our ML framework quantified a mean deltoid volume of 342.2 ± 113.0 cm^3^; however, we observed differences in mean deltoid volume by age and gender, with male patients having a mean deltoid volume between 397.0 to 478.6 cm^3^ and females having a mean deltoid volume between 237.1 to 304.1 cm^3^. Our deltoid volume measurements are similar to the 380.5 ± 157.7 cm^3^ reported by Holzbaur et al. [12], who analyzed the deltoid volume from MRI scans of 10 healthy patients (5M/5F) with a mean age of 28.6 ± 4.5 years (range: 24–37 years) and similar to the of 313.7 ± 77.3 cm^3^ reported by Vidt et al. [13], who analyzed the deltoid volume from MRI scans of 18 elderly patients with a mean age 75.1 ± 4.3 years (range: 66–83 years) [12,13]. Small differences in deltoid volume between studies are likely due to the differences in patient age and gender; specifically, regarding the Holzbaur et al. study [12], our patient cohort was substantially older (70.0 ± 7.9 years, range: 38–92 years), and our results demonstrate that deltoid volume declines between 6–8% per patient age-decade. 

Our ML framework quantified a mean deltoid fatty infiltration of 20.0 ± 9.9%; however, we observed differences in deltoid fat percentage by age, gender, and, most importantly, by CT image kernel, where, specifically, BONE kernel patients had a deltoid fatty infiltration of 13.0 ± 5.6% and FC30 kernel patients had a deltoid fatty infiltration of 28.4 ± 4.6%. Only a few studies quantified deltoid fatty infiltration as a percentage of volume. Vidt et al. analyzed the fat percentage of all shoulder abductors, including the deltoid, from MRI scans of 18 elderly patients and reported an overall fat percentage of 26.5 ± 2.4%, with males (25.6 ± 2.4%) having a slightly lower mean fat percentage than females (27.6 ± 2.2%) [13]. Kälin et al. analyzed the deltoid fatty infiltration from MRI scans of 76 patients (37M/39F) and reported a deltoid fatty infiltration rate of 6.7 ± 2.7% for females and 6.9 ± 3.5% for males [16]. Similarly, Wiater et al. analyzed the deltoid fatty infiltration from MRI scans of 25 rTSA patients and reported a deltoid fatty infiltration rate of 7.9 ± 4.3% [15]. Given that these measurements are all HU-based, differences in fatty infiltration between studies are most likely related to differences in measurement techniques and also in the acquisition and reconstruction protocols of the medical images from which the measurements are derived. Future work is required to further investigate the impact of different HU-based thresholds and the impact of different CT image kernels on the accuracy of fat volume segmentation. 

Both the size/shape and quality of a muscle are important and related to its force production capacity. A larger muscle has a greater cross-sectional area and, therefore, a larger moment arm; as such, it is likely that patients with more muscle volume have improved biomechanics. Specifically related to rTSA, patients with larger deltoid volumes likely have larger deltoid abduction moment arms and greater deltoid efficiency, requiring less deltoid force to elevate the arm. Conversely, rTSA patients with deltoids having high fatty infiltration likely have a reduced force capacity and may have less efficient deltoids, requiring more deltoid force to elevate the arm. These differences in joint loading may have important clinical implications on rTSA complication rates, particularly for acromial and scapular stress fractures, instability, and aseptic loosening. 

A few small studies have suggested that deltoid muscle volume and fatty infiltration impact rTSA clinical outcomes. Greiner et al. quantified deltoid fatty infiltration in 18 rTSA patients and reported that patients with greater deltoid fatty infiltration had significantly lower Constant scores [19]. Wiater et al. quantified deltoid volume in 30 rTSA patients and deltoid fatty infiltration in 25 rTSA patients and reported that deltoid volume significantly correlated to 2-year minimum Constant, ASES, and subjective shoulder value scores and reported that greater deltoid fatty infiltration significantly correlated to decreased 2-year minimum ASES scores [15]. Yoon et al. quantified deltoid volume (normalized by BMI) in 35 rTSA patients with 1-year minimum clinical follow-up and reported that deltoid volume was significantly correlated to the Constant score, forward elevation, and external rotation [20]. McClatchy et al. quantified deltoid volume for a small region of the deltoid in 107 patients from a combination of both MRI and CT images and reported that both deltoid volume and deltoid volume normalized by BMI were significantly (positively) associated with satisfactory levels of forward elevation; additionally, a sub-analysis clarified that preoperative deltoid volume was significantly associated with satisfactory levels of forward elevation for rotator cuff deficient patients but not those with an intact rotator cuff [17].

Our large-scale multi-center analysis of deltoids from 1057 patients has the statistical power to investigate the impact of various 3D deltoid radiomic measures across different age and gender cohorts to provide more valid and generalizable results. We observed that normalized deltoid volume was associated with significantly more preoperative abduction (for both male and female patients), significantly more forward elevation and internal rotation, and a significantly higher SAS score, specifically for male patients. However, regarding 2-year minimum rTSA outcomes, larger deltoid volumes were not associated with more shoulder motion or higher clinical outcome scores for either male or female patients. Our analysis related to the impact of fatty infiltration was less clear, at least for preoperative clinical measures, as the direction of the correlations seems to be partially dependent upon CT image kernel. Female patients with BONE kernel CT images having greater fatty infiltration were associated with significantly more preoperative abduction, external rotation, strength, and a significantly higher global shoulder function score but significantly less preoperative internal rotation; in contrast, female patients with FC30 kernel CT images having greater fatty infiltration were associated with a significantly lower preoperative SAS score. Male patients with FC30 kernel CT images having greater fatty infiltration were associated with significantly less forward elevation. However, 2-year minimum postoperative trends were more consistent, as rTSA patients with either BONE kernel or FC30 kernel CT images who had greater fatty infiltration were associated with significantly less strength and significantly lower Constant and ASES scores. Additionally, patients with BONE kernel CT images having greater fatty infiltration also had significantly less forward elevation and internal rotation and a significantly lower SAS score. 

The relationship between deltoid muscle atrophy and deltoid fatty infiltration is unclear. It may be that these are independent processes, each generally correlated with loss of muscle strength and shoulder function, or it may be that these processes are related. Generally, loss of muscle mass and increased fatty accumulation are thought to be a result of complex interactions of factors, including decreased muscle repair capacity secondary to metabolic changes, increased insulin resistance, higher percentage/redistribution of body fat, decreased physical activity, changes in hormone levels, nutritional deficits, and chronic inflammation [46,47,48]. Future work is required to investigate etiologic pathways and better understand the relationship between deltoid muscle atrophy and deltoid fatty infiltration for each shoulder arthroplasty-related diagnosis. Future work is also required to investigate the impact of intramuscular fat and extramuscular fat, as well as the impact of diffuse vs. localized fat accumulation on clinical outcomes. Ultimately, an improved understanding of these pathways will likely improve treatment decision-making (including the timing of treatment), as it is likely that the quality and size/shape of the shoulder muscles at the time of intervention, as well as their likelihood of progressive degradation, impacts clinical outcomes after aTSA and rTSA. 

This multi-phase study (1. CT image analysis, 2. CT image/clinical data collection and analysis, and 3. the ML model development) has numerous limitations. First, regarding the limitations of our CT image analysis, our deltoid muscle analysis only characterized the overall deltoid muscle volume and did not analyze the characteristics of any individual deltoid muscle segment. Future work is required to divide the deltoid into different functional segments, such as the anterior deltoid, middle deltoid, and posterior deltoid, and/or sub-segments using the seven different intramuscular tendons, as identified by Sakoma et al. [49], and separately analyze the radiomic measures associated with each segment to further investigate the impact on clinical outcomes after aTSA and rTSA. Similarly, future work should compare relative size differences between deltoid muscle segments to identify the impact of any abnormal muscle imbalance on preoperative function or glenoid bone wear patterns and identify the impact of any abnormal muscle imbalance as a risk factor for complications, like instability or aseptic glenoid loosening. Second, we did not analyze the rotator cuff as others [50,51,52,53,54,55,56,57,58] have attempted, or other muscles in the shoulder, like pectoralis, latissimus, or scapular elevators. It is possible that injuries, degeneration, or abnormalities associated with any of these muscles may have some impact on deltoid morphology and function. Future work will deploy a similar CT-based ML framework to analyze the rotator cuff and other shoulder muscles to investigate any such relationships with the deltoid and better understand the impact that each shoulder muscle group has on clinical outcomes after aTSA and rTSA. Third, regarding the radiomic HU-based measurements, we assumed that HU intervals of −190 to −30 accurately characterized fat and HU intervals of −29 to 150 accurately characterized muscle; we acknowledge that this HU threshold likely included some transitional/hybrid tissue in either the bone or muscle segments. Future work should investigate if tissue with HU intervals of −29 to 29 is normal or should be excluded from the muscle/fat segmentation analysis. Additionally, it is likely that the use of a single HU threshold across convolution kernels is not appropriate given the differences we observed in HU distribution between kernels, where, specifically, we observed that some kernels (e.g., BONE kernel) are associated with lower HU fat percentages than other kernels (e.g., FC30 kernel). Kernels with greater noise, like the FC30 images, may interfere with HU threshold calculations of fat, potentially overstating the fatty infiltration quantified with FC30 images. This may explain the surprising lack of significant correlation between patient BMI and fatty infiltration in the FC30 patients, as was observed for male and female patients with BONE images. Furthermore, it is currently unclear if the same attenuation ranges should be used for HU thresholding for patients of different ages, genders, and ethnicities. Fourth, we quantified the deltoid using fourteen different radiomic features (six volumetric-based measurements and eight HU intensity-based measurements); it should be noted that there is a library of additional radiomic measurements that we could have included, and it is possible that some of these additional measurements not analyzed in our study have some predictive capability. Future work is required to assess the validity of these additional measurements and ensure that the most relevant measurements with the greatest contribution to predicting postoperative range of motion, strength, and function are included in the final deployed image-based predictive models. Fifth, even though we identified many deltoid image features that were highly predictive and useful to the ML models, it is unclear exactly why the ML models utilized some features more than others. For example, deltoid shape flatness was the consensus most important feature utilized across all the ML predictive models; however, few differences in preoperative and postoperative clinical outcomes were observed for aTSA or rTSA when stratifying by this image-based measurement. As such, future work should seek to improve the interpretability of these ML predictions and better understand under which conditions these image-based measures are most predictive of clinical outcomes. Given that the F-Score describes the frequency of decision tree splits, it may be that deltoid shape flatness is useful (by itself or in combination with other size/shape and muscle quality measures) to inform the development of a new deltoid morphology classification system that is relevant to aTSA/rTSA clinical outcomes. Future work should attempt a clustering analysis of deltoid measures to identify any relevant relationships/associations. Sixth, our deltoid image-based clinical outcome comparison included patients of multiple different diagnoses, as opposed to a more homogenous cohort. As such, it is unclear from our analysis if these deltoid image features are descriptive of the etiologic mechanisms associated with each of the patient’s various disease diagnoses or if these radiomic measures are merely descriptive of secondary symptoms. Future work is necessary to analyze patient cohorts with homogenous diagnoses to better understand the capability of these measures to better characterize a patient’s diagnosis/pathology on a continuum for each shoulder arthroplasty-related diagnosis. 

Regarding the limitations associated with CT image/clinical data collection, first, our study utilized images from multiple clinical sites, which provided CT images acquired from different CT scanner manufacturers using slightly different imaging protocols (though each CT scan met the minimum requirements specified by ExactechGPS protocol; notably, all CT images were collected within 6 months of the patient’s surgery, the maximum allowable slice thickness was 1.25 mm, and 65% of CT images had a slice thickness of 0.5 or 0.625 mm). While our results demonstrated that different CT image kernels are associated with different radiomic measures, our results are limited because we did not have an equivalent distribution of kernels within our dataset. Future work should harmonize CT-derived metrics using batch effect correction methods to improve measurement reliability [59]. Second, we did not have any longitudinal CT images for any patients, so we were unable to analyze the repeatability of any deltoid measurements. Third, we did not have multiple CT images of any patient from different CT scanner manufacturers and/or CT images with different convolution kernels; therefore, we were unable to compare any radiomic measurement directly for the same patient at equivalent timepoints between CT image types. However, we did separately analyze radiomic measures for the various convolution kernels and identified numerous differences between measurements between kernels. Finally, we did not have CT images of healthy patients for use as a control for our deltoid measurements; future work should obtain CT images of healthy patients and identify baselines for each deltoid image measurement, ideally for patients of similar age as those analyzed in our study. 

Regarding the limitations associated with the ML model development and underlying clinical data, first, the patients in this study were contributed to by 18 different clinical sites, including >25 surgeons, and data from each site/surgeon inevitably contain some bias. As such, the derived models will also contain bias [42,43,60]. To reduce collection bias and input variability, all sites were trained to collect data using standardized data forms, and all completed forms were independently verified before computer-scoring on a secured database. Second, surgeons who contributed clinical data were experienced shoulder specialists who had multiple years of experience with the prosthesis utilized in this study; as such, these predictions may not be translatable to less experienced surgeons or to surgeons who have not completed the learning curve with these devices. Third, our clinical database consists only of patients who elected to undergo shoulder arthroplasty, and those patients are primarily elderly, non-Hispanic, and Caucasians of European descent. For example, we did not collect data on individuals who were candidates for shoulder arthroplasty but elected to forgo surgery due to comorbid illness and financial or personal reasons. Therefore, model predictions may not be representative of the outcomes achieved by patients of different demographics, regions, or ethnicities/races, and model predictions may be biased against patients too sick to safely undergo the procedure or patients whose condition was not sufficiently degenerative to have the procedure. However, recent research by Allen et al. [36] demonstrated that the ML models utilized by Predict+ accurately predict clinical outcomes after aTSA and rTSA for patients of different ethnicities, sexes, and ages. Fourth, our models were developed from a dataset of primary aTSA and primary rTSA patients using one platform shoulder prosthesis, where patients with revisions, humeral fractures, or hemiarthroplasty were excluded; therefore, model predictions may not be appropriate for those excluded indications or other prosthesis types or designs. Fifth, our study utilized one tree-based machine learning technique to construct algorithms that quantify outcomes after shoulder arthroplasty; other techniques, such as deep learning, could achieve better predictive accuracy than XGBoost, as has been shown previously using the Wide and Deep [61] ML technique. Despite these small improvements in accuracy using Wide and Deep, we utilized the XGBoost in our study because its predictions are more interpretable, providing an F-score that identifies the most meaningful parameters used by the model.

## 5. Conclusions

Our ML framework successfully analyzed CT images from 1057 patients and quantified numerous volumetric-based and HU intensity-based deltoid measures, many of which were demonstrated to be relevant to preoperative and postoperative clinical outcomes after aTSA and rTSA. Incorporating these preoperative CT-based deltoid image measurements into our ML models improved the accuracy of the clinical outcome predictions, particularly for abduction and forward elevation after aTSA and rTSA. While future work is required to further refine the ML models and include additional shoulder muscles, like the rotator cuff, our results show promise that the developed ML framework can be used to evolve traditional CT-based preoperative planning software into an evidence-based ML-CDST.

## Figures and Tables

**Table 1:** Definitions of Volumetric-Based and Hounsfield (HU) Intensity-Based Deltoid Image Measurements.

Image Parameter	Type of Image Measurement	Definition
Deltoid Shape Flatness	Volumetric Data	The true flatness of the shape or region of interest is based on spacing between voxels. The less space between voxels, the flatter the object. Value ranges between 1 (non-flat, sphere-like) and 0 (a flat surface or single-slice segmentation).
Normalized Deltoid Volume	Volumetric Data	Deltoid volume normalized or adjusted to the size of patients’ scapula. It is calculated as the ratio of deltoid volume to scapula volume. Larger values imply a larger deltoid.
Normalized Deltoid Atrophy	Volumetric Data	The ratio of individual normalized deltoid volume to the mean normalized deltoid volume across all patients with same age and gender. Values range between 0–1 (if deltoid is smaller than the average deltoid, the atrophy is higher) and more than 1 (if deltoid is larger than average deltoid, the atrophy is lower).
Deltoid Shape Sphericity	Volumetric Data	A measure of the roundness of the shape or the region of interest relative to a sphere. It is the ratio of the surface area of a perfect sphere with the same volume to the surface area of the given object. Values range between 1 (perfect sphere) and 0 (a flat surface).
Max 2D Diameter Row	Volumetric Data	Largest distance between two points in sagittal plane; can be approximated as deltoid length.
Max 2D Diameter Column	Volumetric Data	Largest distance between two points in coronal plane; can be approximated as deltoid width.
Convolution Kernel	Image Protocol	A process used in CT scanners during image reconstruction by reducing blurring and noise captured in the raw data. Different convolution kernels are used to enhance or suppress certain features in the raw image (e.g., muscle or bone). The kernels for the images in the dataset are BONE, FC30, BONEPLUS, B60s, B31s, and [‘I31s, ‘3′].
Skewness of Deltoid Voxels	Hounsfield-Based Data	The asymmetry of the distribution of Hounsfield Unit (HU) values against the average HU of deltoid voxels. The value can be positive or negative depending on tail of the distribution and the average of the distribution.
Deltoid Fat Percentage	Hounsfield-Based Data	The number of deltoid voxels with HU between [−190, −30], representing fat, divided by the numbers of voxels with HU between [−190, 150], representing soft tissue (both muscle and fat). Values range between 0 (0% fat) to 1 (100% fat).
Entropy of Deltoid Voxels	Hounsfield-Based Data	The uncertainty/randomness in the HU values across deltoid voxels. More entropy implies more variation in the values of HU. The value can be any real positive number.
Uniformity of Deltoid Voxels	Hounsfield-Based Data	The homogeneity of Hounsfield unit, where a greater uniformity implies a greater homogeneity or a smaller range of discrete intensity values. Values range between 0 (low uniformity) and 1 (high uniformity).
Mean Deltoid Voxel	Hounsfield-Based Data	The average value of Hounsfield unit in the deltoid voxels.
Root Mean Square Deltoid Voxel	Hounsfield-Based Data	The mean of all the squared Hounsfield unit values. It is another measure of the magnitude of the voxel intensity.
90 percentile Deltoid Voxel	Hounsfield-Based Data	The 90th percentile of Hounsfield unit (voxel intensity) values in the deltoid voxels.
Kurtosis of Deltoid Voxels	Hounsfield-Based Data	A measure of the ‘peakedness’ of the distribution of Hounsfield unit values. A lower kurtosis implies that the peak HU values are near the average HU value, and a high kurtosis implies peak HU values are far from the average HU values.

**Table 2:** Comparison of Volumetric-Based and Hounsfield Intensity-Based Deltoid Image Measurements for Primary aTSA and rTSA Patients Stratified by Gender.

	Deltoid Shape Flatness	Normalized Deltoid Volume	Deltoid Shape Sphericity	Deltoid Fat Percentage	Skewness of Deltoid Voxels	Normalized Deltoid Atrophy	Entropy of Deltoid Voxels	Uniformity of Deltoid Voxels	Max 2D Diameter Column (mm)	Max 2D Diameter Row (mm)	Mean Deltoid Voxel	Kurtosis of Deltoid Voxels	Root Mean Square Deltoid Voxel	90% Deltoid Voxel
**All Patients**	0.469 ± 0.043	3.85 ± 0.64	0.458 ± 0.027	20.0 ± 9.9%	0.14 ± 2.05	1.05 ± 0.16	3.54 ± 0.58	0.114 ± 0.056	161.7 ± 15.8	134.6 ± 20.7	34.9 ± 9.1	24.0 ± 159.5	1038.5 ± 9.4	128.5 ± 39.3
**aTSA, Male**	0.475 ± 0.035	4.17 ± 0.65	0.463 ± 0.024	15.4 ± 8.7%	−0.16 ± 2.02	1.06 ± 0.15	3.43 ± 0.51	0.120 ±0.046	174.7 ± 12.4	147.6 ± 15.2	42.2 ± 7.1	15.8 ± 49.8	1045.0 ± 7.1	127.3 ± 33.4
**aTSA, Female**	0.452 ± 0.038	3.81 ± 0.55	0.459 ± 0.029	19.7 ± 8.1%	0.05 ± 2.11	1.04 ± 0.14	3.54 ± 0.45	0.110 ± 0.043	156.1 ± 12.5	118.7 ± 15.9	33.6 ± 8.2	41.7 ± 259.6	1037.4 ± 10.3	123.5 ± 29.6
**rTSA, Male**	0.487 ± 0.039	3.95 ± 0.62	0.459 ± 0.027	19.5 ± 10.0%	−0.05 ± 1.85	1.04 ± 0.16	3.54 ± 0.63	0.116 ± 0.062	172.0 ± 13.3	149.7 ± 16.5	38.2 ± 7.7	20.9 ± 68.7	1041.7 ± 8.2	133.5 ± 42.3
**rTSA, Female**	0.460 ± 0.045	3.70 ± 0.61	0.455 ± 0.027	21.6 ± 10.1%	0.39 ± 2.15	1.05 ± 0.17	3.56 ± 0.61	0.113 ± 0.057	152.0 ± 11.5	124.0 ± 16.1	30.9 ± 8.6	24.3 ± 191.0	1034.5 ± 8.7	126.1 ± 40.1
***p*-Value (aTSA, Male vs. Female)**	**<0.0001**	**<0.0001**	0.2286	**<0.0001**	0.4347	0.5325	0.0666	0.0725	**<0.0001**	**<0.0001**	**<0.0001**	0.2483	**<0.0001**	0.3338
***p*-Value (rTSA, Male vs. Female)**	**<0.0001**	**<0.0001**	0.0668	**0.0028**	**0.0034**	0.8254	0.6032	0.5744	**<0.0001**	**<0.0001**	**<0.0001**	0.7585	**<0.0001**	**0.0126**
***p*-Value (aTSA, Male vs. rTSA Male)**	**0.0025**	**0.0006**	0.1397	**<0.0001**	0.5648	0.4071	0.0603	0.4273	**0.0400**	0.2069	**<0.0001**	0.4271	**<0.0001**	0.1232
***p*-Value (aTSA, Female vs. rTSA Female)**	0.0564	0.0771	0.2078	0.0541	0.1237	0.9474	0.6698	0.5867	**0.0008**	**0.0013**	**0.0016**	0.4120	**0.0019**	0.4997

**Table 3:** Comparison of Volumetric-Based and Hounsfield Intensity-Based Deltoid Image Measurements for the Most Common Convolution Kernels.

	Deltoid Shape Flatness	Normalized Deltoid Volume	Deltoid Shape Sphericity	Deltoid Fat Percentage	Skewness of Deltoid Voxels	Normalized Deltoid Atrophy	Entropy of Deltoid Voxels	Uniformity of Deltoid Voxels	Max 2D Diameter Column (mm)	Max 2D Diameter Row (mm)	Mean Deltoid Voxel	Kurtosis of Deltoid Voxels	Root Mean Square Deltoid Voxel	90% Deltoid Voxel
**All Kernels**	0.469 ± 0.043	3.85 ± 0.64	0.458 ± 0.027	20.0 ± 9.9%	0.14 ± 2.05	1.05 ± 0.16	3.54 ± 0.58	0.114 ± 0.056	161.7 ± 15.8	134.6 ± 20.7	34.9 ± 9.1	24.0 ± 159.5	1038.5 ± 9.4	128.5 ± 39.3
**Bone (*n* = 448)**	0.466 ± 0.041	3.72 ± 0.57	0.452 ± 0.025	13.0 ± 5.6%	0.09 ± 2.05	1.01 ± 0.14	3.19 ± 0.35	0.139 ± 0.036	161.1 ± 15.4	135.3 ± 20.3	36.2 ± 8.8	24.0 ± 67.5	1037.9 ± 8.6	103.9 ± 18.5
**BonePlus (*n* = 160)**	0.468 ± 0.043	3.87 ± 0.60	0.460 ± 0.025	26.6 ± 8.7%	−0.06 ± 2.08	1.06 ± 0.15	4.04 ± 0.52	0.079 ± 0.031	159.3 ± 16.8	130.9 ± 20.6	34.3 ± 9.3	21.1 ± 62.0	1040.6 ± 10.3	164.6 ± 48.0
**FC30 (*n* = 305)**	0.470 ± 0.046	4.10 ± 0.65	0.464 ± 0.029	28.4 ± 4.6%	0.43 ± 2.34	1.10 ± 0.16	3.94 ± 0.15	0.075 ± 0.009	163.3 ± 15.5	136.2 ± 21.6	33.4 ± 9.2	32.1 ± 281.9	1038.2 ± 9.9	152.0 ± 13.7
***p*-Value (Bone vs. BonePlus)**	0.6470	**0.0037**	**0.0004**	**<0.0001**	0.4342	**0.0005**	**<0.0001**	**<0.0001**	0.3929	**0.0209**	**0.0249**	0.6339	**0.0021**	**<0.0001**
***p*-Value (Bone vs. FC30)**	0.2102	**<0.0001**	**<0.0001**	**<0.0001**	**0.0336**	**<0.0001**	**<0.0001**	**<0.0001**	0.0514	0.5326	**<0.0001**	0.5595	0.7562	**<0.0001**
***p*-Value (BonePlus vs. FC30)**	0.6073	**0.0002**	0.1026	**0.0044**	**0.0263**	**0.0203**	**0.0020**	0.0836	**0.0260**	**0.0109**	0.3282	0.6265	**0.0155**	**<0.0001**

**Table 4:** Comparison of Deltoid Volume for Patients of Different Genders and Ages at the Time of Surgery.

	Deltoid Volume (cm^3^)	Percent Difference from Preceding Age Cohort	Deltoid Volume Normalized by Scapular Bone Volume	Percent Difference from Preceding Age Cohort	Deltoid Volume Normalized by Patient Height	Percent Difference from Preceding Age Cohort	Deltoid Fat %
**Male, <60 yrs**	478.6 ± 102.6	NA	4.51 ± 0.71	NA	6.86 ± 1.38	NA	18.2%
**Male, 60–70 yrs**	456.4 ± 86.7	−4.6%	4.13 ± 0.60	−8.4%	6.59 ± 1.18	−3.9%	17.9%
**Male, 70–80 yrs**	424.4 ± 76.7	−7.0%	3.86 ± 0.54	−6.5%	6.13 ± 1.02	−7.0%	18.2%
**Male, >80 yrs**	397.0 ± 56.6	−6.5%	3.62 ± 0.60	−6.2%	5.70 ± 0.76	−7.0%	20.4%
**Female, <60 yrs**	304.1 ± 48.5	NA	4.18 ± 0.55	NA	4.78 ± 0.73	NA	22.9%
**Female, 60–70 yrs**	278.1 ± 58.5	−8.5%	3.87 ± 0.60	−7.4%	4.33 ± 0.82	−9.4%	22.0%
**Female, 70–80 yrs**	254.0 ± 52.3	−8.7%	3.64 ± 0.55	−5.9%	4.01 ± 0.76	−7.4%	21.1%
**Female, >80 yrs**	237.1 ± 47.3	−6.7%	3.42 ± 0.63	−6.0%	3.81 ± 0.72	−5.0%	18.9%

**Table 5:** Impact of Normalized Deltoid Volume on Preoperative Clinical Outcome Measures for Male and Female Patients.

	Deltoid Volume (cm^3^)	Active Abduction	Active Forward Elevation	IR Score	Active External Rotation	Strength (Max Weight)	VAS Pain	Global Shoulder Function	Constant	ASES	SAS
**Male, Normalized Deltoid Volume < 4.0**	392.9 ± 60.4	83.8 ± 36.2	96.8 ± 36.0	3.4 ± 1.7	22.5 ± 23.1	4.8 ± 5.4	5.6 ± 2.2	4.5 ± 1.9	44.4 ± 15.6	43.0 ± 16.0	50.1 ± 11.2
**Male, Normalized Deltoid Volume > 4.0**	491.8 ± 78.9	98.5 ± 39.2	110.4 ± 37.1	3.8 ± 1.7	25.6 ± 21.0	5.5 ± 5.5	5.8 ± 2.2	4.7 ± 2.1	47.4 ± 15.9	43.2 ± 15.7	52.7 ± 11.3
***p*-Value**	**<0.0001**	**<0.0001**	**<0.0001**	**0.0108**	0.1341	0.1983	0.2388	0.3510	0.0685	0.8719	**0.0160**
**Female, Normalized Deltoid Volume < 4.0**	245.0 ± 46.4	77.0 ± 33.4	92.9 ± 35.6	3.3 ± 1.8	19.8 ± 21.5	2.8 ± 2.9	6.4 ± 2.1	4.0 ± 2.0	38.1 ± 13.9	35.7 ± 14.5	45.9 ± 11.4
**Female, Normalized Deltoid Volume > 4.0**	305.1 ± 54.7	83.7 ± 39.5	96.3 ± 38.1	3.2 ± 1.9	23.2 ± 20.3	2.4 ± 3.2	6.4 ± 2.2	4.1 ± 2.1	38.7 ± 15.1	35.0 ± 15.3	46.8 ± 12.5
***p*-Value**	**<0.0001**	**0.0405**	0.3060	0.8284	0.0800	0.1843	0.6823	0.5453	0.6771	0.6275	0.3981

**Table 6:** Impact of Normalized Deltoid Volume on 2-year Minimum Clinical Outcome Measures for aTSA and rTSA Patients Stratified by Gender.

	Active Abduction	Active Forward Elevation	IR Score	Active External Rotation	Strength (Max Weight)	VAS Pain	Global Shoulder Function	Constant	ASES	SAS
**aTSA Male, Normalized Deltoid Volume < 4.0**	150.5 ± 18.6	155.9 ± 29.6	5.0 ± 1.7	58.4 ± 15.9	12.1 ± 4.8	1.0 ± 1.7	8.8 ± 1.7	77.8 ± 10.2	89.2 ± 14.6	83.2 ± 9.8
**aTSA Male, Normalized Deltoid Volume > 4.0**	147.5 ± 25.2	157.4 ± 19.0	5.4 ± 1.1	61.6 ± 14.1	12.4 ± 5.7	1.6 ± 2.3	8.4 ± 1.8	73.8 ± 15.1	83.1 ± 19.0	83.0 ± 11.3
***p*-Value**	0.5748	0.7843	0.3233	0.3508	0.7842	0.1920	0.3058	0.2216	0.1200	0.9619
**aTSA Female, Normalized Deltoid Volume < 4.0**	143.6 ± 31.5	156.4 ± 22.9	5.3 ± 1.2	57.3 ± 15.8	6.9 ± 3.6	0.8 ± 1.3	8.8 ± 1.6	72.3 ± 11.7	89.4 ± 13.6	84.2 ± 9.6
**aTSA Female, Normalized Deltoid Volume > 4.0**	135.2 ± 41.2	145.0 ± 33.9	5.1 ± 1.4	57.2 ± 18.4	8.2 ± 4.5	1.3 ± 2.2	8.9 ± 1.3	73.9 ± 16.0	85.2 ± 19.5	80.7 ± 14.3
***p*-Value**	0.3283	0.0847	0.5532	0.9789	0.2212	0.1816	0.7181	0.6516	0.2855	0.2281
**rTSA Male, Normalized Deltoid Volume < 4.0**	126.4 ± 25.1	142.4 ± 21.0	4.0 ± 1.7	40.2 ± 19.0	10.9 ± 4.9	0.9 ± 1.8	8.4 ± 2.0	71.1 ± 13.1	85.9 ± 16.4	76.4 ± 10.3
**rTSA Male, Normalized Deltoid Volume > 4.0**	128.8 ± 29.0	145.8 ± 18.2	4.3 ± 1.6	41.0 ± 16.4	10.8 ± 5.6	1.5 ± 2.3	8.3 ± 1.9	73.7 ± 12.6	82.0 ± 20.3	76.5 ± 10.6
***p*-Value**	0.5004	0.2632	0.2027	0.7827	0.9041	**0.0457**	0.7484	0.2240	0.1441	0.9740
**rTSA Female, Normalized Deltoid Volume < 4.0**	117.4 ± 27.6	140.3 ± 27.9	4.7 ± 1.7	41.8 ± 18.3	6.6 ± 3.8	1.4 ± 2.0	8.2 ± 1.9	66.3 ± 14.1	81.7 ± 17.8	76.8 ± 11.3
**rTSA Female, Normalized Deltoid Volume > 4.0**	127.8 ± 29.0	137.8 ± 26.0	4.5 ± 1.8	42.9 ± 18.5	6.8 ± 4.1	1.9 ± 2.4	7.4 ± 2.2	66.5 ± 13.9	76.5 ± 20.2	73.2 ± 12.2
***p*-Value**	**0.0091**	0.5184	0.3442	0.6814	0.8071	0.0795	**0.0034**	0.9245	**0.0373**	**0.0379**

**Table 7:** Relationship between Deltoid Fat Percentage and Patient Demographics and Comorbidities for Male and Female Patients Stratified by the BONE Kernel and FC30 Kernel CT Image Cohorts.

	Weight	BMI	Previous Shoulder Surgery %	OA Diagnosis	RCT Diagnosis	CTA Diagnosis	No Comorbidities	Inflammatory Arthritis	Hypertension	Heart Disease	Diabetes
**Male, Deltoid Fat % <13% (BONE)**	193.3 ± 34.4	28.6 ± 4.6	25.3%	65.7%	6.0%	32.8%	45.5%	1.5%	42.5%	14.2%	7.5%
**Male, Deltoid Fat % >13% (BONE)**	200.3 ± 34.3	30.1 ± 5.1	31.5%	66.7%	13.0%	29.6%	29.6%	1.9%	57.4%	22.2%	20.4%
***p* Value**	0.2123	**0.0479**	0.3967	0.8970	0.1105	0.6716	**0.0452**	0.8600	0.0650	0.1806	**0.0109**
**Female, Deltoid Fat % <13% (BONE)**	149.4 ± 33.2	26.1 ± 4.9	22.8%	66.7%	15.8%	32.5%	43.9%	0.9%	46.5%	5.3%	6.1%
**Female, Deltoid Fat % >13% (BONE)**	177.5 ± 36.9	31.1 ± 6.0	25.3%	65.7%	16.4%	19.9%	24.0%	6.9%	58.9%	11.6%	14.4%
***p*-Value**	**<0.0001**	**<0.0001**	0.6375	0.8779	0.8884	**0.0206**	**0.0006**	**0.0175**	**0.0467**	0.0727	**0.0335**
**Male, Deltoid Fat % <28% (FC30)**	213.1 ± 43.2	30.0 ± 5.5	32.9%	64.9%	11.7%	25.5%	20.2%	3.2%	61.7%	20.2%	17.0%
**Male, Deltoid Fat % >28% (FC30)**	192.9 ± 28.4	28.7 ± 4.8	45.3%	52.8%	13.2%	39.6%	7.6%	7.6%	73.6%	26.4%	26.4%
***p*-Value**	**0.0028**	0.1323	0.1407	0.1528	0.7909	0.0760	**0.0427**	0.2367	0.1462	0.3903	0.1772
**Female, Deltoid Fat % <28% (FC30)**	175.6 ± 45.1	30.7 ± 7.8	35.9%	46.2%	15.3%	38.5%	23.1%	7.7%	69.2%	10.3%	15.4%
**Female, Deltoid Fat % >28% (FC30)**	181.7 ± 41.3	31.5 ± 6.5	31.1%	55.5%	10.9%	27.7%	9.2%	13.5%	71.4%	17.6%	21.8%
***p*-Value**	0.4393	0.5536	0.5804	0.3151	0.4606	0.2085	**0.0241**	0.3409	0.7947	0.2753	0.3866

**Table 8:** Impact of Deltoid Fat Percentage on Preoperative Clinical Outcome Measures for Male and Female Patients Stratified by the BONE Kernel and FC30 Kernel CT Image Cohorts.

	Deltoid Volume (cm^3^)	Active Abduction	Active Forward Elevation	IR Score	Active External Rotation	Strength (Max Weight)	VAS Pain	Global Shoulder Function	Constant	ASES	SAS
**Male, Deltoid Fat % <13% (BONE)**	437.5 ± 81.6	94.7 ± 39.7	112.3 ± 37.9	4.0 ± 1.5	21.1 ± 24.2	7.9 ± 5.4	5.7 ± 2.2	4.5 ± 2.3	51.1 ± 14.9	44.1 ± 16.7	53.2 ± 10.8
**Male, Deltoid Fat % >13% (BONE)**	409.8 ± 84.1	97.1 ± 42.6	109.3 ± 39.2	3.6 ± 1.7	26.1 ± 24.6	7.9 ± 5.8	5.8 ± 2.3	5.0 ± 1.7	52.2 ± 14.2	45.5 ± 15.9	52.7 ± 10.1
***p*-Value**	**0.0377**	0.7150	0.6205	0.0718	0.1984	0.9830	0.8559	0.0962	0.6504	0.6084	0.7924
**Female, Deltoid Fat % <13% (BONE)**	245.4 ± 47.7	74.9 ± 25.4	106.8 ± 31.7	4.0 ± 1.7	14.2 ± 23.2	3.6 ± 2.5	6.6 ± 1.8	3.3 ± 1.9	41.3 ± 13.8	34.8 ± 13.9	47.0 ± 11.1
**Female, Deltoid Fat % >13% (BONE)**	261.4 ± 48.3	88.0 ± 37.6	98.3 ± 36.5	3.1 ± 1.7	22.5 ± 20.0	4.8 ± 3.3	6.6 ± 1.9	4.3 ± 2.1	43.0 ± 14.7	35.4 ± 12.8	46.4 ± 11.0
***p*-Value**	**0.0068**	**0.0017**	0.0536	**<0.0001**	**0.0024**	**0.0019**	0.7333	**<0.0001**	0.3597	0.6927	0.6696
**Male, Deltoid Fat % <28% (FC30)**	485.5 ± 85.1	88.1 ± 36.6	97.4 ± 37.0	3.2 ± 1.8	22.9 ± 23.1	2.7 ± 4.4	5.7 ± 2.0	4.6 ± 1.9	41.8 ± 16.0	42.3 ± 13.0	48.8 ± 11.8
**Male, Deltoid Fat % >28% (FC30)**	402.4 ± 75.1	75.9 ± 35.4	80.9 ± 36.2	3.1 ± 1.7	23.1 ± 15.1	0.9 ± 1.8	5.6 ± 2.5	4.1 ± 2.0	37.1 ± 14.6	39.7 ± 15.4	47.3 ± 10.4
***p*-Value**	**<0.0001**	0.0540	**0.0104**	0.7009	0.9659	**0.0072**	0.6643	0.2137	0.0931	0.2783	0.4335
**Female, Deltoid Fat % <28% (FC30)**	288.6 ± 82.0	78.7 ± 30.3	89.3 ± 32.8	3.2 ± 2.0	21.2 ± 20.1	0.7 ± 1.7	6.0 ± 2.8	4.2 ± 2.1	35.4 ± 13.1	37.4 ± 18.8	46.0 ± 13.3
**Female, Deltoid Fat % >28% (FC30)**	280.0 ± 65.1	73.1 ± 32.5	80.7 ± 32.0	2.6 ± 1.8	20.2 ± 16.3	0.6 ± 1.3	6.8 ± 1.9	4.0 ± 2.0	31.4 ± 11.2	32.3 ± 13.2	41.5 ± 10.1
***p*-Value**	0.5036	0.3432	0.1500	0.0557	0.7548	0.5040	0.0697	0.6616	0.0784	0.0683	**0.0351**

**Table 9:** Impact of Deltoid Fat Percentage on 2-year Minimum Clinical Outcome Measures for aTSA and rTSA Patients Stratified by the BONE Kernel and FC30 Kernel CT Image Cohorts.

	Active Abduction	Active Forward Elevation	IR Score	Active External Rotation	Strength (Max Weight)	VAS Pain	Global Shoulder Function	Constant	ASES	SAS
**aTSA, Deltoid Fat % <13% (BONE)**	153.0 ± 19.0	163.9 ± 11.3	5.6 ± 1.1	60.6 ± 13.6	12.7 ± 4.7	0.9 ± 1.4	9.0 ± 1.1	79.5 ± 11.1	90.8 ± 11.1	86.6 ± 6.8
**aTSA, Deltoid Fat % >13% (BONE)**	157.0 ± 24.9	162.1 ± 20.5	4.9 ± 1.4	55.7 ± 17.1	8.1 ± 3.6	0.8 ± 1.7	9.2 ± 1.1	72.6 ± 12.1	90.7 ± 14.1	83.9 ± 9.1
***p*-Value**	0.3996	0.5941	**0.0148**	0.1332	**<0.0001**	0.7460	0.4546	**0.0083**	0.9664	0.1570
**rTSA, Deltoid Fat % <13% (BONE)**	121.3 ± 25.6	153.7 ± 21.3	5.0 ± 1.4	41.1 ± 18.0	10.3 ± 5.0	1.3 ± 2.1	8.2 ± 1.9	74.0 ± 12.6	83.6 ± 17.7	78.9 ± 10.8
**rTSA, Deltoid Fat >13% (BONE)**	122.4 ± 28.8	140.1 ± 22.3	3.6 ± 1.9	38.7 ± 21.8	7.7 ± 4.0	1.8 ± 2.2	7.7 ± 2.2	61.6 ± 12.6	76.7 ± 19.8	72.7 ± 11.2
***p*-Value**	0.7914	**<0.0001**	**<0.0001**	0.4291	**0.0006**	0.1264	0.1534	**<0.0001**	**0.0175**	**0.0014**
**aTSA, Deltoid Fat % <28% (FC30)**	137.1 ± 14.2	140.7 ± 13.7	5.5 ± 0.7	57.1 ± 11.7	10.5 ± 7.1	2.1 ± 2.5	8.5 ± 1.4	75.4 ± 11.1	79.1 ± 17.3	80.2 ± 9.8
**aTSA, Deltoid Fat % >28% (FC30)**	104.2 ± 35.4	121.9 ± 33.8	4.8 ± 1.8	48.1 ± 14.7	3.2 ± 3.3	2.3 ± 2.8	8.1 ± 2.4	61.6 ± 17.9	76.7 ± 24.2	73.0 ± 17.1
***p*-Value**	**0.0036**	0.0662	0.1674	0.0869	**0.0046**	0.8366	0.5791	**0.0267**	0.7507	0.2034
**rTSA, Deltoid Fat % <28% (FC30)**	124.3 ± 25.8	135.8 ± 20.6	4.4 ± 1.6	40.1 ± 14.5	9.7 ± 5.6	1.2 ± 1.8	8.6 ± 1.8	73.4 ± 13.1	83.0 ± 16.0	76.2 ± 10.1
**rTSA, Deltoid Fat >28% (FC30)**	120.4 ± 27.7	128.8 ± 23.9	4.4 ± 1.8	38.5 ± 15.4	5.9 ± 3.7	1.8 ± 2.4	7.9 ± 2.2	66.6 ± 13.1	76.4 ± 20.4	73.0 ± 10.9
***p*-Value**	0.4316	0.0950	0.8507	0.5450	**<0.0001**	0.1443	0.0626	**0.0073**	**0.0433**	0.1149

**Table 10:** Comparison of the Mean Absolute Error (MAE) Associated with Two Different Machine Learning Models (Image-Based vs. No-Image Data) to Predict Clinical Outcomes at 1 year, 2–3 years, and 3–5 years after aTSA and rTSA.

Clinical Outcome Prediction ALL (aTSA, rTSA)	1 yr MAE of Predict+ w/o Deltoid Image Data (aTSA, rTSA)	1 yr MAE of Predict+ with Volumetric and Hounsfield-Based Image Data	Percent Difference in MAE (1 yr)	2–3 yr MAE of Predict+ w/o Deltoid Image Data (aTSA, rTSA)	2–3 yr MAE of Predict+ with Volumetric and Hounsfield-Based Image Data	Percent Difference in MAE (2–3 yrs)	3–5 yr MAE of Predict+ w/o Deltoid Image Data (aTSA, rTSA)	3–5 yr MAE of Predict+ with Volumetric and Hounsfield-Based Image Data	Percent Difference in MAE (3–5 yrs)
**Abduction**	19.7 (18.8, 20.0)	18.5 (18.1, 18.6)	6.3% (3.6%, 7.0%)	18.8 (17.8, 19.2)	17.8 (17.5, 17.9)	5.3% (1.7%, 6.8%)	19.1 (16.5, 20.1)	17.2 (13.9, 18.6)	9.6% (16.1%, 7.1%)
**External Rotation**	12.3 (12.9, 12.1)	12.3 (12.7, 12.1)	0.1% (1.4%, −0.5%)	12.9 (12.9, 12.9)	13.2 (12.5, 13.5)	−2.4% (2.8%, −4.4%)	13.1 (13.1, 13.1)	12.5 (13.5, 12.1)	5.1% (−2.8%, 7.8%)
**Forward Elevation**	16.4 (15.7, 16.6)	15.8 (15.2, 16.0)	3.6% (2.8%, 3.8%)	15.1 (14.8, 15.2)	13.8 (13.2, 14.1)	8.5% (10.8%, 7.5%)	14.3 (11.9, 15.3)	14.0 (11.5, 14.9)	1.8% (3.0%, 2.3%)
**Internal Rotation Score**	1.26 (1.14, 1.30)	1.30 (1.18, 1.35)	−3.5% (−3.3%, −3.9%)	1.14 (0.95, 1.22)	1.18 (0.95, 1.27)	−3.2% (0.1%, −3.8%)	1.10 (1.02, 1.15)	1.07 (0.99, 1.11)	3.1% (2.6%, 3.7%)
**ASES**	12.2 (11.6, 12.4)	12.3 (11.5, 12.6)	−1.0% (1.1%, −1.6%)	12.8 (10.9, 13.6)	12.5 (11.2, 12.9)	2.7% (−2.2%, 4.6%)	10.7 (8.3, 11.5)	10.7 (8.5, 11.4)	−0.3% (−2.8%, 1.0%)
**Constant**	8.4 (7.8, 8.6)	8.6 (8.6, 8.6)	−2.0% (−9.0%, 0.3%)	9.5 (9.9, 9.3)	9.3 (9.1, 9.4)	1.6% (8.2%, −1.1%)	7.8 (8.0, 7.8)	7.7 (7.5, 7.7)	2.3% (6.0%, 0.6%)
**SAS**	7.8 (8.0, 7.7)	8.1 (8.5, 7.9)	−3.1% (−5.8%, −2.0%)	8.1 (8.5, 7.9)	7.6 (7.6, 7.5)	6.7% (10.3%, 4.9%)	6.9 (6.0, 7.2)	6.6 (6.4, 6.8)	4.4% (−6.3%, 6.6%)
**Global Shoulder Function**	1.36 (1.25, 1.39)	1.35 (1.24, 1.38)	0.6% (0.6%, 0.9%)	1.32 (1.11, 1.40)	1.36 (1.14, 1.45)	−3.6% (−2.8%, −4.2%)	1.27 (0.82, 1.46)	1.36 (0.85, 1.58)	−7.5% (−3.8%, −8.0%)
**VAS Pain**	1.40 (1.32, 1.42)	1.45 (1.38, 1.47)	−3.6% (−4.6%, −3.2%)	1.54 (1.29, 1.65)	1.48 (1.27, 1.56)	4.0% (1.5%, 5.2%)	1.17 (1.09, 1.19)	1.15 (1.00, 1.20)	1.3% (8.0%, −1.2%)

**Table 11:** Classification Prediction Performance Associated with Two Different Machine Learning Models (Image-Based vs. No-Image Data) to Predict aTSA and rTSA Clinical Improvement at 2–3 Years Follow-Up Greater Than the MCID Threshold for Multiple Different Outcome Measures.

All (aTSA, rTSA)	ML Model	Active Abduction	Active Forward Elevation	IR Score	Active External Rotation	VAS Pain	Global Shoulder Function	Constant	ASES	SAS
**MCID**		7.85 (11.3, 4.4)	12.85 (17.2, 8.5)	0.6 (0.6, −0.1)	6.05 (9.5, 2.6)	−1.3 (−1.3, −1.3)	1.1 (1.1, 1.1)	5.8 (8.6, 3.0)	12.7 (14.2, 11.2)	6.65 (8.4, 4.9)
**Precision, %**	Non-image	92.1% (88.8%, 93.4%)	93.5% (91.8%, 93.7%)	79.6% (85.9%, 88.9%)	83.9% (88.3%, 85.3%)	90.8% (93.0%, 89.9%)	90.9% (90.6%, 90.9%)	91.5% (82.3%, 94.4%)	91.8% (94.7%, 91.4%)	93.2% (92.8%, 95.2%)
	Image model	92.5% (89.3%, 94.7%)	93.2% (92.9%, 95%)	79.5% (90.3%, 86.4%)	83.6% (87.9%, 85.2%)	92.1% (94.3%, 91.2%)	90.9% (88.2%, 92.1%)	91.5% (85.7%, 94.6%)	93.2% (93.7%, 93.7%)	93.2% (93.3%, 94.3%)
**Recall, %**	Non-image	95.2% (93.2%, 95.9%)	93.5% (88.3%, 93.9%)	79.6% (81.1%, 84.8%)	90.6% (89.5%, 92.2%)	95.5% (96.9%, 95.0%)	96.5% (96.1%, 96.6%)	95.8% (88.2%, 98.9%)	97.1% (98.6%, 96.0%)	98.3% (98.7%, 98.7%)
	Image model	92.8% (91.5%, 94.7%)	94.5% (85.9%, 95.6%)	82.2% (85.4%, 83.8%)	90.5% (90.2%, 91.7%)	95.3% (96.8%, 94.7%)	95.9% (97.1%, 95.3%)	95.5% (90.4%, 98.1%)	96.1% (98.4%, 95.4%)	98.2% (99.5%, 98.3%)
**Accuracy, %**	Non-image	89.1% (85.4%, 90.6%)	89.6% (84.7%, 89.6%)	75.2% (77.3%, 79.6%)	80.0% (81.7%, 81.9%)	88.2% (90.8%, 87.1%)	89.0% (88.4%, 89.2%)	89.8% (79.1%, 94.2%)	89.8% (93.8%, 88.4%)	92.2% (92.1%, 94.3%)
	Image model	87.6% (84%, 90.8%)	90.1% (84.1%, 92.1%)	76.6% (82.5%, 77.4%)	80.2% (82.4%, 81.7%)	89.1% (91.8%, 88.0%)	88.6% (87.2%, 89.1%)	89.4% (82.1%, 93.7%)	90.3% (92.6%, 90.1%)	92.1% (93.2%, 93.1%)
**AUROC**	Non-image	0.761 (0.753, 0.767)	0.837 (0.806, 0.81)	0.742 (0.735, 0.728)	0.696 (0.695, 0.694)	0.729 (0.702, 0.729)	0.743 (0.733, 0.745)	0.822 (0.733, 0.855)	0.655 (0.74, 0.62)	0.726 (0.719, 0.766)
	Image model	0.761 (0.727, 0.806)	0.834 (0.804, 0.848)	0.753 (0.802, 0.701)	0.718 (0.704, 0.722)	0.752 (0.696, 0.756)	0.747 (0.716, 0.764)	0.809 (0.767, 0.854)	0.682 (0.692, 0.688)	0.717 (0.710, 0.744)

**Table 12:** Classification Prediction Performance Associated with Two Different Machine Learning Models (Image-Based vs. No-Image Data) to Predict aTSA and rTSA Clinical Improvement at 2–3 Years Follow-Up Greater Than the SCB Threshold for Multiple Different Outcome Measures.

All (aTSA, rTSA)	ML Model	Active Abduction	Active Forward Elevation	IR Score	Active External Rotation	VAS Pain	Global Shoulder Function	Constant	ASES	SAS
**SCB**		25.6 (31.1, 20.1)	29.85 (36.7, 23.0)	0.25 (1.1, 0.6)	12.55 (16.4, 8.7)	−2.9 (−3.1, −2.7)	2.8 (3.0, 2.6)	17.5 (20.4, 14.6)	30.95 (33.2, 28.7)	16.75 (19.1, 14.4)
**Precision, %**	Non-image	84.3% (80.5%, 85.0%)	93.1% (89.9%, 92.1%)	76.5% (80.4%, 76.7%)	81.4% (84.4%, 84.3%)	88.9% (83.2%, 89.3%)	89.4% (71.6%, 89%)	87.9% (77.2%, 92.0%)	84.2% (87.6%, 86.7%)	86.3% (83.7%, 87.4%)
	Image model	86.6% (79.5%, 88.8%)	92.9% (90.9%, 92.3%)	75.9% (80.3%, 74.9%)	80.6% (84.9%, 84.4%)	90.8% (87.3%, 89.8%)	88.7% (74.2%, 89.1%)	87.0% (78.7%, 92.7%)	84.7% (85.6%, 86.9%)	87.7% (84.9%, 89.1%)
**Recall, %**	Non-image	92.0% (87.2%, 94.8%)	91.4% (90.9%, 95.2%)	87.5% (84.2%, 78.7%)	85.4% (87.2%, 87.2%)	89% (93.4%, 89.9%)	85.5% (85.2%, 88.6%)	89.5% (76.4%, 92.8%)	87.9% (85.2%, 89.5%)	91.0% (87.3%, 93.3%)
	Image model	92.3% (88.5%, 94.8%)	91.9% (91.9%, 95.8%)	86.8% (87.8%, 80.6%)	84.5% (88.5%, 87.5%)	87.6% (89.5%, 89.2%)	85.4% (86.5%, 87%)	86.7% (76.7%, 91.7%)	86.6% (86.4%, 88.1%)	92.2% (90.2%, 92.2%)
**Accuracy, %**	Non-image	83.0% (80.6%, 84.2%)	89.3% (90.0%, 90.4%)	76.1% (82.6%, 74.4%)	77.2% (80.4%, 79.1%)	83.1% (81.7%, 84.4%)	81.7% (74.9%, 83.0%)	85.5% (79.3%, 88.8%)	79.2% (79.7%, 81.5%)	82.2% (77.9%, 84.1%)
	Image model	85.1% (80.9%, 87.6%)	89.4% (91.4%, 90.9%)	75.5% (82.0%, 74.4%)	76.6% (82.1%, 79.7%)	83.4% (82.3%, 84.2%)	81.2% (76.8%, 82.0%)	82.8% (78.9%, 88.4%)	78.9% (79.6%, 80.8%)	84.1% (80.2%, 84.7%)
**AUROC**	Non-image	0.779 (0.793, 0.757)	0.879 (0.900, 0.862)	0.733 (0.825, 0.738)	0.73 (0.774, 0.726)	0.762 (0.737, 0.786)	0.782 (0.746, 0.773)	0.840 (0.790, 0.855)	0.720 (0.746, 0.725)	0.725 (0.689, 0.718)
	Image model	0.814 (0.802, 0.821)	0.877 (0.912, 0.863)	0.728 (0.819, 0.737)	0.731 (0.795, 0.747)	0.781 (0.757, 0.789)	0.776 (0.762, 0.770)	0.808 (0.786, 0.851)	0.724 (0.742, 0.724)	0.747 (0.698, 0.736)

**Table 13:** Comparison of the Relative Feature Importance Ranking of Each CT-based Deltoid Image Parameter to Predict 2–3-year Clinical Outcomes after aTSA and rTSA.

F-Score Rank, Reciprocal Fusion Rank Score	Deltoid Shape Flatness	Normalized Deltoid Volume	Deltoid Shape Sphericity	Deltoid Fat Percentage	Skewness of Deltoid Voxels	Normalized Deltoid Atrophy	Entropy of Deltoid Voxels	Uniformity of Deltoid Voxels	Max 2D Diameter Column	Max 2D Diameter Row	Mean Deltoid Voxel	Kurtosis of Deltoid Voxels	Root Mean Square Deltoid Voxel	90% Deltoid Voxel	Kernel
**Abduction**	2, 1	9, 4	7, 3	13, 7	5, 2	14, 5	16, 6	21, 8	46, 38	36, 30	42, 35	45, 37	44, 36	51, 43	41, 42
**External Rotation**	8, 1	10, 2	12, 6	7, 5	11, 3	16, 4	22, 7	32, 12	39, 34	35, 29	50, 38	40, 36	38, 33	45, 39	49, 47
**Forward Elevation**	7, 4	4, 2	5, 3	3, 5	2, 1	13, 6	14, 7	28, 9	42, 34	43, 35	48, 41	45, 37	44, 36	50, 42	49, 47
**Internal Rotation Score**	2, 1	6, 2	11, 3	7, 4	12, 5	17, 6	18, 7	29, 10	41, 31	45, 36	43, 34	48, 39	46, 37	49, 41	50, 48
**ASES**	2, 1	9, 5	4, 2	13, 7	7, 3	12, 4	16, 6	29, 9	37, 32	51, 42	40, 33	52, 38	57, 44	61, 48	64, 62
**Constant**	3, 2	2, 1	9, 4	10, 7	11, 5	12, 3	14, 6	27, 9	42, 37	43, 35	45, 38	46, 36	50, 42	58, 46	62, 56
**SAS**	5, 1	8, 3	11, 6	6, 5	10, 4	12, 2	18, 7	26, 11	42, 33	44, 35	43, 38	45, 39	47, 36	50, 43	51, 46
**Global Shoulder Function**	10, 5	3, 1	7, 3	8, 6	6, 2	13, 4	18, 7	25, 9	35, 30	39, 35	36, 32	41, 37	45, 38	48, 39	52, 48
**VAS Pain**	6, 2	11, 4	8, 3	12, 7	16, 5	10, 1	17, 6	29, 10	43, 37	41, 33	38, 31	42, 36	47, 39	50, 41	51, 48
**Average F-Score Rank Order**	5.0	6.9	8.2	8.8	8.9	13.2	17.0	27.3	40.8	41.9	42.8	44.9	46.4	51.3	52.1
**Average Reciprocal Fusion Rank Score Order**	2.0	2.7	3.7	5.9	3.3	3.9	6.6	9.7	34.0	34.4	35.6	37.2	37.9	42.4	49.3

**Table 14:** Comparison of the Top 10 Preoperative Model Inputs (by F-Score Ranking) Used to Predict 2–3-year Clinical Outcomes after aTSA and rTSA.

F-Score Ranking of Preoperative Inputs	Abduction	External Rotation	Forward Elevation	Internal Rotation Score	ASES	Constant	SAS	Global Shoulder Function	VAS Pain
1 (Most Used Feature)	Abduction	Abduction	Abduction	Abduction	Native Glenoid Retroversion	Native Glenoid Retroversion	Abduction	Abduction	Abduction
2	Deltoid Shape Flatness	External Rotation	Skewness of Deltoid Voxels	Deltoid Shape Flatness	Deltoid Shape Flatness	Normalized Deltoid Volume	Native Glenoid Retroversion	Native Glenoid Retroversion	External Rotation
3	Native Glenoid Retroversion	Native Glenoid Retroversion	Deltoid Fat Percentage	VAS Pain	External Rotation	Deltoid Shape Flatness	Composite ROM Score	Normalized Deltoid Volume	Native Glenoid Retroversion
4	SAS	SAS	Normalized Deltoid Volume	Native Glenoid Retroversion	Deltoid Shape Sphericity	Abduction	External Rotation	Composite ROM Score	VAS Pain
5	Skewness of Deltoid Voxels	Age at Surgery	Deltoid Shape Sphericity	External Rotation	Abduction	External Rotation	Deltoid Shape Flatness	External Rotation	Forward Elevation
6	Composite ROM Score	Forward Elevation	Native Glenoid Retroversion	Normalized Deltoid Volume	Forward Elevation	SAS	Deltoid Fat Percentage	Skewness of Deltoid Voxels	Deltoid Shape Flatness
7	Deltoid Shape Sphericity	Deltoid Fat Percentage	Deltoid Shape Flatness	Deltoid Fat Percentage	Skewness of Deltoid Voxels	Composite ROM Score	SAS	Deltoid Shape Sphericity	Composite ROM Score
8	Age at Surgery	Deltoid Shape Flatness	SAS	Composite ROM Score	Composite ROM Score	Forward Elevation	Normalized Deltoid Volume	Deltoid Fat Percentage	Deltoid Shape Sphericity
9	Normalized Deltoid Volume	Composite ROM Score	Age at Surgery	Forward Elevation	Normalized Deltoid Volume	Deltoid Shape Sphericity	Forward Elevation	Forward Elevation	SAS
10	External Rotation	Normalized Deltoid Volume	External Rotation	SAS	SAS	Deltoid Fat Percentage	Skewness of Deltoid Voxels	Deltoid Shape Flatness	Normalized Deltoid Atrophy

**Table 15:** Pearson Correlation for a Selection of Deltoid Image Parameters to Preoperative and 2-Year Minimum Postoperative Outcome Measures for aTSA and rTSA. Moderate Correlations (>±0.3) or Higher Highlighted for Emphasis.

aTSA, rTSA	Deltoid Shape Flatness	Normalized Deltoid Volume	Deltoid Shape Sphericity	Deltoid Fat Percentage	Skewness of Deltoid Voxels	Normalized Deltoid Atrophy	Entropy of Deltoid Voxels
**Preop Abduction**	0.166, 0.018	0.091, 0.202	−0.004, 0.144	−0.076, −0.033	−0.001, −0.045	0.073, 0.170	0.017, 0.033
**Preop Forward Elevation**	0.247, −0.027	0.130, 0.153	0.014, 0.109	−0.212, −0.196	−0.048, −0.021	0.094, 0.122	−0.097, −0.118
**Preop IR Score**	0.187, 0.186	0.103, 0.091	−0.097, −0.024	−0.141, −0.248	−0.002, 0.042	0.077, 0.080	−0.045, −0.172
**Preop Ext. Rotation**	0.138, 0.065	0.149, 0.091	0.005, 0.012	−0.053, 0.096	0.055, 0.001	0.183, 0.106	0.046, 0.144
**Preop Max Weight in Hand**	0.138, −0.022	0.061, 0.029	−0.057, −0.052	−0.440, −0.289	−0.052, −0.118	−0.039, −0.028	−0.285, −0.173
**Preop VAS Pain**	−0.090, −0.139	−0.036, −0.033	0.050, −0.004	0.049, −0.035	−0.066, −0.028	−0.063, −0.028	−0.054, −0.103
**Preop Global Shoulder Function**	0.175, −0.005	0.110, 0.069	−0.034, 0.019	0.058, 0.061	0.070, −0.055	0.126, 0.086	0.178, 0.130
**Preop Constant**	0.216, 0.045	0.028, 0.124	−0.145, 0.034	−0.334, −0.233	−0.057, −0.043	−0.002, 0.088	−0.165, −0.117
**Preop ASES**	0.173, 0.142	0.055, 0.066	−0.080, −0.018	−0.088, −0.034	0.052, 0.029	0.064, 0.064	0.065, 0.056
**Preop SAS**	0.263, 0.150	0.168, 0.147	−0.059, 0.028	−0.151, −0.119	0.045, 0.009	0.162, 0.119	0.016, 0.000
**2 yr Min Abduction**	0.031, 0.005	0.061, 0.156	0.052, 0.092	−0.186, 0.017	−0.063, −0.090	0.035, 0.075	−0.056, 0.069
**2 yr Min Forward Elevation**	0.067, 0.048	0.002, 0.053	0.033, 0.011	−0.301, −0.281	−0.033, −0.097	−0.040, −0.007	−0.179, −0.245
**2 yr Min IR Score**	0.031, 0.080	−0.034, 0.058	−0.107, −0.049	−0.213, −0.133	0.008, 0.033	−0.002, 0.079	−0.162, −0.139
**2 yr Min Ext. Rotation**	0.065, 0.004	−0.018, 0.037	−0.073, −0.020	−0.227, −0.040	−0.072, 0.005	−0.047, 0.031	−0.156, 0.015
**2 yr Min Max Weight in Hand**	0.196, 0.027	0.082, 0.126	−0.001, 0.055	−0.395, −0.230	0.001, −0.104	−0.040, 0.018	−0.253, −0.127
**2 yr Min VAS Pain**	0.053, −0.026	0.228, 0.054	0.064, 0.039	0.188, 0.001	−0.020, 0.036	0.196, 0.077	0.173, −0.031
**2 yr Min Global Shoulder Function**	−0.047, 0.032	−0.119, −0.025	−0.040, −0.039	−0.153, −0.019	−0.035, −0.073	−0.116, −0.052	−0.096, −0.002
**2 yr Min Constant**	−0.017, 0.054	−0.124, 0.120	−0.052, 0.021	−0.333, −0.160	0.005, −0.087	−0.127, 0.049	−0.276, −0.128
**2 yr Min ASES**	−0.052, 0.046	−0.190, −0.045	−0.064, −0.053	−0.220, −0.047	−0.026, −0.054	−0.177, −0.083	−0.175, −0.006
**2 yr Min SAS**	−0.002, 0.043	−0.125, 0.001	−0.054, −0.066	−0.319, −0.134	−0.052, −0.046	−0.124, −0.029	−0.241, −0.105

**Table 16:** Impact of Deltoid Shape Flatness on Preoperative Clinical Outcome Measures for Male and Female Patients.

	Deltoid Volume (cm^3^)	Active Abduction	Active Forward Elevation	IR Score	Active External Rotation	Strength (Max Weight)	VAS Pain	Global Shoulder Function	Constant	ASES	SAS
**Male, Deltoid Flatness <0.47**	435.7 ± 86.1	88.6 ± 36.7	102.4 ± 33.8	3.2 ± 1.8	23.2 ± 22.5	5.6 ± 5.4	5.7 ± 2.2	4.6 ± 1.9	46.3 ± 15.3	43.5 ± 15.6	50.1 ± 11.0
**Male, Deltoid Flatness >0.47**	443.5 ± 85.2	92.4 ± 39.4	103.9 ± 38.9	3.7 ± 1.6	24.5 ± 21.9	4.8 ± 5.5	5.7 ± 2.2	4.7 ± 2.1	45.5 ± 16.1	42.9 ± 16.0	52.1 ± 11.5
***p*-Value**	0.3393	0.2999	0.6743	**0.0014**	0.5549	0.1651	0.9161	0.6707	0.6194	0.6988	0.0619
**Female, Deltoid Flatness <0.47**	263.2 ± 56.5	80.5 ± 34.6	94.8 ± 34.5	3.1 ± 1.8	20.0 ± 20.4	2.8 ± 3.1	6.5 ± 2.0	4.1 ± 2.1	37.8 ± 14.1	34.3 ± 14.2	45.3 ± 11.6
**Female, Deltoid Flatness >0.47**	263.7 ± 56.1	76.6 ± 37.0	92.5 ± 39.4	3.6 ± 1.8	22.2 ± 22.5	2.6 ± 2.8	6.1 ± 2.3	3.9 ± 2.1	38.9 ± 14.6	37.5 ± 15.5	47.5 ± 11.8
***p*-Value**	0.9234	0.2109	0.4665	**0.0013**	0.2419	0.5253	**0.0151**	0.3196	0.4159	**0.0147**	**0.0358**

**Table 17:** Impact of Deltoid Shape Flatness on 2-year Minimum Clinical Outcome Measures for aTSA and rTSA Patients Stratified by Gender.

	Active Abduction	Active Forward Elevation	IR Score	Active External Rotation	Strength (Max Weight)	VAS Pain	Global Shoulder Function	Constant	ASES	SAS
**aTSA Male, Deltoid Flatness <0.47**	147.7 ± 21.2	151.9 ± 30.5	5.0 ± 1.6	59.5 ± 16.7	10.7 ± 4.3	1.5 ± 2.2	8.4 ± 1.7	73.0 ± 11.8	85.4 ± 17.0	81.5 ± 9.9
**aTSA Male, Deltoid Flatness >0.47**	149.4 ± 23.8	159.9 ± 17.7	5.4 ± 1.3	60.9 ± 13.7	13.3 ± 5.7	1.3 ± 2.1	8.6 ± 1.8	77.1 ± 14.1	85.7 ± 18.0	84.1 ± 11.0
***p*-Value**	0.7515	0.1496	0.2121	0.6998	0.0501	0.6707	0.4915	0.2230	0.9424	0.3432
**aTSA Female, Deltoid Flatness <0.47**	139.2 ± 35.7	150.7 ± 29.1	5.2 ± 1.4	55.2 ± 17.4	7.2 ± 4.1	1.0 ± 1.8	8.8 ± 1.5	72.6 ± 13.9	87.3 ± 16.6	82.2 ± 12.4
**aTSA Female, Deltoid Flatness >0.47**	145.0 ± 33.1	157.7 ± 21.6	5.5 ± 0.8	62.5 ± 13.3	7.7 ± 3.7	0.8 ± 1.3	9.0 ± 1.6	73.4 ± 11.4	89.6 ± 13.4	85.2 ± 7.9
***p*-Value**	0.5122	0.3121	0.2914	0.0800	0.6070	0.6757	0.6480	0.8175	0.5640	0.3268
**rTSA Male, Deltoid Flatness <0.47**	129.5 ± 23.6	147.5 ± 19.2	4.2 ± 1.7	41.8 ± 17.6	12.5 ± 5.8	1.1 ± 2.0	8.6 ± 1.5	76.6 ± 11.7	86.6 ± 18.2	78.4 ± 9.2
**rTSA Male, Deltoid Flatness >0.47**	126.7 ± 24.7	142.4 ± 19.9	4.1 ± 1.6	40.0 ± 18.0	10.2 ± 4.8	1.3 ± 2.1	8.2 ± 2.1	70.4 ± 13.0	82.9 ± 18.4	75.6 ± 10.8
***p*-Value**	0.4880	0.1230	0.5874	0.5429	**0.0120**	0.5226	0.1921	**0.0054**	0.1990	0.1164
**rTSA Female, Deltoid Flatness <0.47**	122.2 ± 29.0	139.9 ± 28.1	4.6 ± 1.9	43.8 ± 17.7	7.2 ± 4.1	1.4 ± 2.1	8.0 ± 2.0	67.4 ± 14.3	80.9 ± 18.1	76.6 ± 11.9
**rTSA Female, Deltoid Flatness >0.47**	118.2 ± 27.6	139.2 ± 26.3	4.8 ± 1.6	39.8 ± 18.9	6.0 ± 3.5	1.7 ± 2.3	7.8 ± 2.1	65.1 ± 13.5	78.8 ± 19.6	74.5 ± 11.5
***p*-Value**	0.2873	0.8574	0.4916	0.0942	**0.0401**	0.2249	0.5118	0.2474	0.3715	0.1995

**Table 18:** Comparison of the Mean Absolute Error (MAE) Associated with Two Different Machine Learning Models (Automated Image-Based with No Surgeon Range of Motion Measurements or Patient Subjective Inputs vs. No-Image Data) to Predict Clinical Outcomes at 1 year, 2–3 years, and 3–5 years after aTSA and rTSA.

Clinical Outcome Prediction	1 yr MAE of Predict+ w/o Deltoid Image Data (aTSA, rTSA)	1 yr MAE of Predict+ with Image Data but No Objective ROM Data and No Patient Subjective Data	Percent Difference in MAE (1 yr)	2–3 yr MAE of Predict+ w/o Deltoid Image Data (aTSA, rTSA)	2–3 yr MAE of Predict+ with Image Data but No Objective ROM Data and No Patient Subjective Data	Percent Difference in MAE (2–3 yrs)	3–5 yr MAE of Predict+ w/o Deltoid Image Data (aTSA, rTSA)	3–5 yr MAE of Predict+ with Image Data but No Objective ROM Data and No Patient Subjective Data	Percent Difference in MAE (3–5 yrs)
**Abduction**	19.7 (18.8, 20.0)	19.7 (19.2, 19.8)	0.3% (−2.1%, 1.0%)	18.8 (17.8, 19.2)	19.3 (19.8, 19.2)	−3.0% (−10.2%, 0.2%)	19.1 (16.5, 20.1)	18.1 (15.8, 18.8)	5.6% (4.4%, 6.7%)
**External Rotation**	12.3 (12.9, 12.1)	13.5 (14.0, 13.4)	−9.2% (−7.8%, −9.6%)	12.9 (12.9, 12.9)	13.0 (12.5, 13.2)	−1.2% (3.1%, −2.4%)	13.1 (13.1, 13.1)	13.7 (14.7, 13.2)	−3.9% (−10.8%, −0.4%)
**Forward Elevation**	16.4 (15.7, 16.6)	15.8 (15.3, 15.9)	4.1% (2.5%, 4.5%)	15.1 (14.8, 15.2)	14.2 (14.7, 14.1)	6.1% (0.7%, 7.6%)	14.3 (11.9, 15.3)	13.5 (11.3, 14.3)	6.0% (4.7%, 6.9%)
**Internal Rotation Score**	1.26 (1.14, 1.30)	1.30 (1.21, 1.33)	−3.0% (−6.2%, −2.7%)	1.14 (0.95, 1.22)	1.23 (0.97, 1.33)	−7.4% (−2.2%, −8.1%)	1.10 (1.02, 1.15)	1.18 (1.09, 1.22)	−5.9% (−6.3%, −5.4%)
**ASES**	12.2 (11.6, 12.4)	12.4 (12.3, 12.5)	−2.0% (−5.5%, −0.5%)	12.8 (10.9, 13.6)	12.7 (11.3, 13.3)	0.7% (−3.3%, 1.9%)	10.7 (8.3, 11.5)	9.8 (7.7, 10.5)	9.3% (7.6%, 9.4%)
**Constant**	8.4 (7.8, 8.6)	8.9 (9.5, 8.8)	−5.7% (−17.1%, −1.5%)	9.5 (9.9, 9.3)	9.4 (9.8, 9.2)	0.8% (0.8%, 0.5%)	7.8 (8.0, 7.8)	7.8 (8.0, 7.8)	−0.1% (−0.5%, −0.8%)
**SAS**	7.8 (8.0, 7.7)	8.0 (8.9, 7.8)	−2.9% (−10.0%, −0.4%)	8.1 (8.5, 7.9)	8.1 (8.1, 8.0)	0.5% (4.8%, −1.3%)	6.9 (6.0, 7.2)	6.8 (7.0, 6.7)	2.3% (−13.7%, 8.3%)
**Global Shoulder Function**	1.36 (1.25, 1.39)	1.35 (1.26, 1.38)	0.7% (−0.8%, 0.9%)	1.32 (1.11, 1.40)	1.36 (1.13, 1.45)	−3.4% (−1.9%, −3.8%)	1.27 (0.82, 1.46)	1.33 (0.91, 1.48)	−4.8% (−10.2%, −1.6%)
**VAS Pain**	1.40 (1.32, 1.42)	1.51 (1.45, 1.53)	−7.5% (−8.8%, −7.2%)	1.54 (1.29, 1.65)	1.49 (1.27, 1.58)	3.7% (1.3%, 4.4%)	1.17 (1.09, 1.19)	1.19 (1.14, 1.22)	−2.3% (−4.2%, −2.2%)

## Data Availability

Data are contained within the article.

## References

[1] Jobin C.M., Brown G.D., Bahu M.J., Gardner T.R., Bigliani L.U., Levine W.N., Ahmad C.S. (2012). Reverse total shoulder arthroplasty for cuff tear arthropathy: The clinical effect of deltoid lengthening and center of rotation medialization. J. Shoulder Elb. Surg..

[2] Lädermann A., Walch G., Denard P.J., Collin P., Sirveaux F., Favard L., Edwards T.B., Kherad O., Boileau P. (2013). Reverse shoulder arthroplasty in patients with pre-operative impairment of the deltoid muscle. Bone Jt. J..

[3] Schwartz D.G., Cottrell B.J., Teusink M.J., Clark R.E., Downes K.L., Tannenbaum R.S., Frankle M.A. (2014). Factors that predict postoperative motion in patients treated with reverse shoulder arthroplasty. J. Shoulder Elb. Surg..

[4] Otis J.C., Jiang C.C., Wickiewicz T.L., Peterson M.G., Warren R.F., Santner T.J. (1994). Changes in the moment arms of the rotator cuff and deltoid muscles with abduction and rotation. J. Bone Jt. Surg. Am..

[5] Kuechle D.K., Newman S.R., Itoi E., Morrey B.F., An K.N. (1997). Shoulder muscle moment arms during horizontal flexion and elevation. J. Shoulder Elb. Surg..

[6] Roche C.P. (2022). Reverse Shoulder Arthroplasty Biomechanics. J. Funct. Morphol. Kinesiol..

[7] Roche C., Crosby L. (2013). Kinematics and Biomechanics of Reverse Total Shoulder Arthroplasty. AAOS Orthopaedic Knowledge Update.

[8] Roche C.P., Hamilton M.A., Diep P., Wright T.W., Flurin P.-H., Zuckerman J.D., Routman H.D. (2015). Optimizing Deltoid Efficiency with Reverse Shoulder Arthroplasty Using a Novel Inset Center of Rotation Glenosphere Design. Bull. Hosp. Jt. Dis..

[9] Hamilton M.A., Diep P., Roche C., Flurin P.H., Wright T.W., Zuckerman J.D., Routman H. (2015). Effect of reverse shoulder design philosophy on muscle moment arms. J. Orthop. Res..

[10] Roche C.P., Diep P., Hamilton M., A Crosby L., Flurin P.-H., Wright T.W., Zuckerman J.D., Routman H.D. (2013). Impact of inferior glenoid tilt, humeral retroversion, bone grafting, and design parameters on muscle length and deltoid wrapping in reverse shoulder arthroplasty. Bull. Hosp. Jt. Dis..

[11] Routman H.D., Flurin P.-H., Wright T.W., Zuckerman J.D., Hamilton M.A., Roche C.P. (2015). Reverse Shoulder Arthroplasty Prosthesis Design Classification System. Bull. Hosp. Jt. Dis..

[12] Holzbaur K.R., Murray W.M., Gold G.E., Delp S.L. (2007). Upper limb muscle volumes in adult subjects. J. Biomech..

[13] Vidt M.E., Daly M., Miller M.E., Davis C.C., Marsh A.P., Saul K.R. (2012). Characterizing upper limb muscle volume and strength in older adults: A comparison with young adults. J. Biomech..

[14] Meyer D.C., Rahm S., Farshad M., Lajtai G., Wieser K. (2013). Deltoid muscle shape analysis with magnetic resonance imaging in patients with chronic rotator cuff tears. BMC Musculoskelet. Disord..

[15] Wiater B.P., Koueiter D.M., Maerz T., Moravek J.E., Yonan S., Marcantonio D.R., Wiater J.M. (2015). preoperative deltoid size and fatty infiltration of the deltoid and rotator cuff correlate to outcomes after reverse total shoulder arthroplasty. Clin. Orthop. Relat. Res..

[16] Kälin P.S., Crawford R.J., Marcon M., Manoliu A., Bouaicha S., Fischer M.A., Ulbrich E.J. (2018). Shoulder muscle volume and fat content in healthy adult volunteers: Quantification with DIXON MRI to determine the influence of demographics and handedness. Skelet. Radiol..

[17] McClatchy S.G., Heise G.M., Mihalko W.M., Azar F.M., A Smith R., Witte D.H., Stanfill J.G., Throckmorton T.W., Brolin T.J. (2022). Effect of deltoid volume on range of motion and patient-reported outcomes following reverse total shoulder arthroplasty in rotator cuff-intact and rotator cuff-deficient conditions. Shoulder Elb..

[18] Nakazawa K., Manaka T., Hirakawa Y., Ito Y., Iio R., Oi N., Nakamura H. (2023). Reliability and validity of a new deltoid muscle area measurement method after reverse shoulder arthroplasty. JSES Int..

[19] Greiner S.H., Back D.A., Herrmann S., Perka C., Asbach P. (2010). Degenerative changes of the deltoid muscle have impact on clinical outcome after reversed total shoulder arthroplasty. Arch. Orthop. Trauma Surg..

[20] Yoon J.P., Seo A., Kim J.J., Lee C.-H., Baek S.-H., Kim S.Y., Jeong E.T., Oh K.-S., Chung S.W. (2017). Deltoid muscle volume affects clinical outcome of reverse total shoulder arthroplasty in patients with cuff tear arthropathy or irreparable cuff tears. PLoS ONE.

[21] van Griethuysen J.J.M., Fedorov A., Parmar C., Hosny A., Aucoin N., Narayan V., Beets-Tan R.G.H., Fillion-Robin J.-C., Pieper S., Aerts H.J.W.L. (2017). Computational Radiomics System to Decode the Radiographic Phenotype. Cancer Res..

[22] Aubrey J., Esfandiari N., Baracos V.E., Buteau F.A., Frenette J., Putman C.T., Mazurak V.C. (2014). Measurement of skeletal muscle radiation attenuation and basis of its biological variation. Acta Physiol..

[23] Lorensen W.E., Cline H.E. (1987). Marching cubes: A high resolution 3D surface construction algorithm. Proceedings of the 14th Annual Conference on Computer Graphics and Interactive Techniques (SIGGRAPH ‘87).

[24] Rajabzadeh-Oghaz H., Elwell J., Kumar V., Mabrouk L., Daviller C., Berry D., Singh A., Polakovic S., Schoch B., Roche C. Machine-Learning Model for Quantification of Deltoid Characteristics. Proceedings of the 2024 Orthopedic Research Society.

[25] Tang Y., Yang D., Li W., Roth H.R., Landman B., Xu D., Nath V., Hatamizadeh A. Self-supervised pre-training of swin transformers for 3d medical image analysis. Proceedings of the IEEE/CVF Conference on Computer Vision and Pattern Recognition.

[26] Kumar V., Roche C., Overman S., Simovitch R., Flurin P.-H., Wright T., Zuckerman J., Routman H., Teredesai A. (2021). Using machine learning to predict clinical outcomes after shoulder arthroplasty with a minimal feature set. J. Shoulder Elb. Surg..

[27] Simmons C., DeGrasse J., Polakovic S., Aibinder W., Throckmorton T., Noerdlinger M., Papandrea R., Trenhaile S., Schoch B., Gobbato B. (2023). Initial clinical experience with a predictive clinical decision support tool for anatomic and reverse total shoulder arthroplasty. Eur. J. Orthop. Surg. Traumatol..

[28] Roche C., Kumar V., Overman S., Simovitch R., Flurin P.-H., Wright T., Routman H., Teredesai A., Zuckerman J. (2021). Validation of a machine learning–derived clinical metric to quantify outcomes after total shoulder arthroplasty. J. Shoulder Elb. Surg..

[29] Flurin P.-H., Marczuk Y., Janout M., Wright T.W., Zuckerman J., Roche C.P. (2013). Comparison of outcomes using anatomic and reverse total shoulder arthroplasty. Bull. NYU Hosp. Jt. Dis..

[30] Simovitch R., Flurin P.-H., Wright T., Zuckerman J.D., Roche C.P. (2018). Quantifying success after total shoulder arthroplasty: The minimal clinically important difference. J. Shoulder Elb. Surg..

[31] Kumar V., Schoch B.S., Allen C., Overman S., Teredesai A., Aibinder W., Parsons M., Watling J., Ko J.K., Gobbato B. (2022). Using machine learning to predict internal rotation after anatomic and reverse total shoulder arthroplasty. J. Shoulder Elb. Surg..

[32] Simovitch R., Flurin P.-H., Wright T., Zuckerman J.D., Roche C.P. (2018). Quantifying success after total shoulder arthroplasty: The substantial clinical benefit. J. Shoulder Elb. Surg..

[33] Kumar V., Roche C.M., Overman S., Simovitch R., Flurin P.-H., Wright T., Zuckerman J., Routman H.D., Teredesai A. (2020). What Is the Accuracy of Three Different Machine Learning Techniques to Predict Clinical Outcomes After Shoulder Arthroplasty?. Clin. Orthop. Relat. Res..

[34] Kumar V., Roche C., Overman S., Simovitch R., Flurin P.-H., Wright T., Zuckerman J., Routman H., Teredesai A. (2021). Use of machine learning to assess the predictive value of 3 commonly used clinical measures to quantify outcomes after total shoulder arthroplasty. Semin. Arthroplast. JSES.

[35] Kumar V., Allen C., Overman S., Teredesai A., Simovitch R., Flurin P.-H., Wright T., Zuckerman J., Routman H., Roche C. (2022). Development of a predictive model for a machine learning–derived shoulder arthroplasty clinical outcome score. Semin. Arthroplast. JSES.

[36] Allen C., Kumar V., Elwell J., Overman S., Schoch B.S., Aibinder W., Parsons M., Watling J., Ko J.K., Gobbato B. (2023). Evaluating the Fairness and Accuracy of Machine Learning–Based Predictions of Clinical Outcomes after Anatomic and Reverse Total Shoulder Arthroplasty. J. Shoulder Elb. Surg..

[37] Aibinder W., Schoch B., Parsons M., Watling J., Ko J.K., Gobbato B., Throckmorton T., Routman H., Fan W., Simmons C. (2021). Risk factors for complications and revision surgery after anatomic and reverse total shoulder arthroplasty. J. Shoulder Elb. Surg..

[38] Simmons C.S., Roche C., Schoch B.S., Parsons M., Aibinder W.R. (2022). Surgeon confidence in planning total shoulder arthroplasty improves after consulting a clinical decision support tool. Eur. J. Orthop. Surg. Traumatol..

[39] Chen T., Guestrin C. Xgboost: A scalable tree boosting system. Proceedings of the 22nd Acm Sigkdd International Conference on Knowledge Discovery and Data Mining.

[40] Torlay L., Perrone-Bertolotti M., Thomas E., Baciu M. (2017). Machine learning–XGBoost analysis of language networks to classify patients with epilepsy. Brain Inform..

[41] Cormack G.V., Clarke C.L., Buettcher S. Reciprocal rank fusion outperforms condorcet and individual rank learning methods. Proceedings of the 32nd International ACM SIGIR Conference on Research and Development in Information Retrieval.

[42] Ahmad M.A., Eckert C., Teredesai A. Interpretable Machine Learning in Healthcare. Proceedings of the 2018 IEEE International Conference on Healthcare Informatics (ICHI).

[43] Lipton Z.C. (2016). The mythos of model interpretability. arXiv.

[44] Parsons M., Greene A., Polakovic S., Byram I., Cheung E., Jones R., Papandrea R., Youderian A., Wright T., Flurin P.-H. (2020). Assessment of surgeon variability in preoperative planning of reverse total shoulder arthroplasty: A quantitative comparison of 49 cases planned by 9 surgeons. J. Shoulder Elb. Surg..

[45] Parsons M., Greene A., Polakovic S., Rohrs E., Byram I., Cheung E., Jones R., Papandrea R., Youderian A., Wright T. (2020). Intersurgeon and intrasurgeon variability in preoperative planning of anatomic total shoulder arthroplasty: A quantitative comparison of 49 cases planned by 9 surgeons. J. Shoulder Elb. Surg..

[46] Siparsky P.N., Kirkendall D.T., Garrett W.E. (2014). Muscle Changes in Aging: Understanding Sarcopenia. Sports Health.

[47] Volpi E., Nazemi R., Fujita S. (2004). Muscle tissue changes with aging. Curr. Opin. Clin. Nutr. Metab. Care.

[48] Hamrick M.W., McGee-Lawrence M.E., Frechette D.M. (2016). Fatty Infiltration of Skeletal Muscle: Mechanisms and Comparisons with Bone Marrow Adiposity. Front. Endocrinol..

[49] Sakoma Y., Sano H., Shinozaki N., Itoigawa Y., Yamamoto N., Ozaki T., Itoi E. (2011). Anatomical and functional segments of the deltoid muscle. J. Anat..

[50] Beeler S., Ek E.T., Gerber C. (2013). A comparative analysis of fatty infiltration and muscle atrophy in patients with chronic rotator cuff tears and suprascapular neuropathy. J. Shoulder Elb. Surg..

[51] Vidt M.E., Santago A.C., Tuohy C.J., Poehling G.G., Freehill M.T., Kraft R.A., Marsh A.P., Hegedus E.J., Miller M.E., Saul K.R. (2016). Assessments of Fatty Infiltration and Muscle Atrophy From a Single Magnetic Resonance Image Slice Are Not Predictive of 3-Dimensional Measurements. Arthrosc. J. Arthrosc. Relat. Surg..

[52] Terrier A., Ston J., Dewarrat A., Becce F., Farron A. (2017). A semi-automated quantitative CT method for measuring rotator cuff muscle degeneration in shoulders with primary osteoarthritis. Orthop. Traumatol. Surg. Res..

[53] Matsumura N., Oguro S., Okuda S., Jinzaki M., Matsumoto M., Nakamura M., Nagura T. (2017). Quantitative assessment of fatty infiltration and muscle volume of the rotator cuff muscles using 3-dimensional 2-point Dixon magnetic resonance imaging. J. Shoulder Elb. Surg..

[54] Lee D., Hong K.-T., Lee W., Khil E.K., Lee G.Y., Choi J.-A., Song Y. (2020). Threshold-based quantification of fatty degeneration in the supraspinatus muscle on MRI as an alternative method to Goutallier classification and single-voxel MR spectroscopy. BMC Musculoskelet. Disord..

[55] Taghizadeh E., Truffer O., Becce F., Eminian S., Gidoin S., Terrier A., Farron A., Büchler P. (2021). Deep learning for the rapid automatic quantification and characterization of rotator cuff muscle degeneration from shoulder CT datasets. Eur. Radiol..

[56] Puzzitiello R.N., Moverman M.A., Menendez M.E., Hart P.-A., Kirsch J., Jawa A. (2021). Rotator cuff fatty infiltration and muscle atrophy do not impact clinical outcomes after reverse total shoulder arthroplasty for glenohumeral osteoarthritis with intact rotator cuff. J. Shoulder Elb. Surg..

[57] Wallenberg R.B., Belzer M.L., Ramsey D.C., Opel D.M., Berkson M.D., Gundle K.R., Nagy M.L., Boucher R.J., McCarron J.A. (2022). MRI-based 3-dimensional volumetric assessment of fatty infiltration and muscle atrophy in rotator cuff tears. J. Shoulder Elb. Surg..

[58] Werthel J.-D., de Casson F.B., Walch G., Gaudin P., Moroder P., Sanchez-Sotelo J., Chaoui J., Burdin V. (2022). Three-dimensional muscle loss assessment: A novel computed tomography–based quantitative method to evaluate rotator cuff muscle fatty infiltration. J. Shoulder Elb. Surg..

[59] Ligero M., Jordi-Ollero O., Bernatowicz K., Garcia-Ruiz A., Delgado-Muñoz E., Leiva D., Mast R., Suarez C., Sala-Llonch R., Calvo N. (2021). Minimizing acquisition-related radiomics variability by image resampling and batch effect correction to allow for large-scale data analysis. Eur. Radiol..

[60] Obermeyer Z., Emanuel E.J. (2016). Predicting the future—Big data, machine learning, and clinical medicine. N. Engl. J. Med..

[61] Zheng Z., Yang Y., Niu X., Dai H.-N., Zhou Y. (2018). Wide and deep convolutional neural networks for electricity-theft detection to secure smart grids. IEEE Trans. Ind. Inform..

